# Harnessing diverse hybrid integration for bridging trans-scale multi-dimensional fiber-chip data transmission and processing

**DOI:** 10.1038/s41377-026-02194-9

**Published:** 2026-03-12

**Authors:** Kang Li, Guofeng Yan, Kangrui Wang, Chengkun Cai, Min Yang, Guangze Wu, Weike Zhao, Yingying Peng, Yaocheng Shi, Daoxin Dai, Jian Wang

**Affiliations:** 1 Wuhan National Laboratory for Optoelectronics and School of Optical and Electronic Information, Huazhong University of Science and Technology, Wuhan, Hubei China; 2 Hubei Optical Fundamental Research Center, Wuhan, Hubei China; 3 Optics Valley Laboratory, Hubei, Wuhan, Hubei China; 4 State Key Laboratory for Modern Optical Instrumentation, Center for Optical & Electromagnetic Research, College of Optical Science and Engineering, International Research Center for Advanced Photonics, Zhejiang University, Zijingang Campus, Hangzhou, China

**Keywords:** Fibre optics and optical communications, Integrated optics

## Abstract

Optical communications have emerged as a promising solution for high-speed modern communication systems and built an important infrastructure for the global information superhighway. Although recent efforts to enhance optical communications have penetrated from long-distance fiber-optic to ultra-short-reach chip-scale data transmission, “Trans-Scale” high-capacity data transmission remains great challenges. In addition to data transmission, data processing is also of great importance for flexible data management in optical communication systems. However, a “Digital Divide” (capacity gap) exists between high-capacity data transmission in fiber links and low-speed data processing at network nodes, hindering the flourishing development of optical communications. Here, we implement “Trans-Scale” high-capacity bridging between few-mode fiber and silicon multimode waveguide using a diverse hybrid integrated coupler, which includes a 3D silica fs-laser direct writing photonic chip and a 2D silicon photonic integrated circuit. On this basis, we leverage a large-scale silicon reconfigurable optical add-drop multiplexer (ROADM) with over 2000 elements to construct a multi-dimensional fiber-chip system, enabling 192-channel (3 modes, 2 polarizations, 32 wavelengths) and 20-Tbit/s trans-scale multi-dimensional data transmission and processing. This demonstration provides a superior trans-scale architecture for multi-dimensional data transmission and processing in next-generation optical communications.

## Introduction

**Fig. 1:**
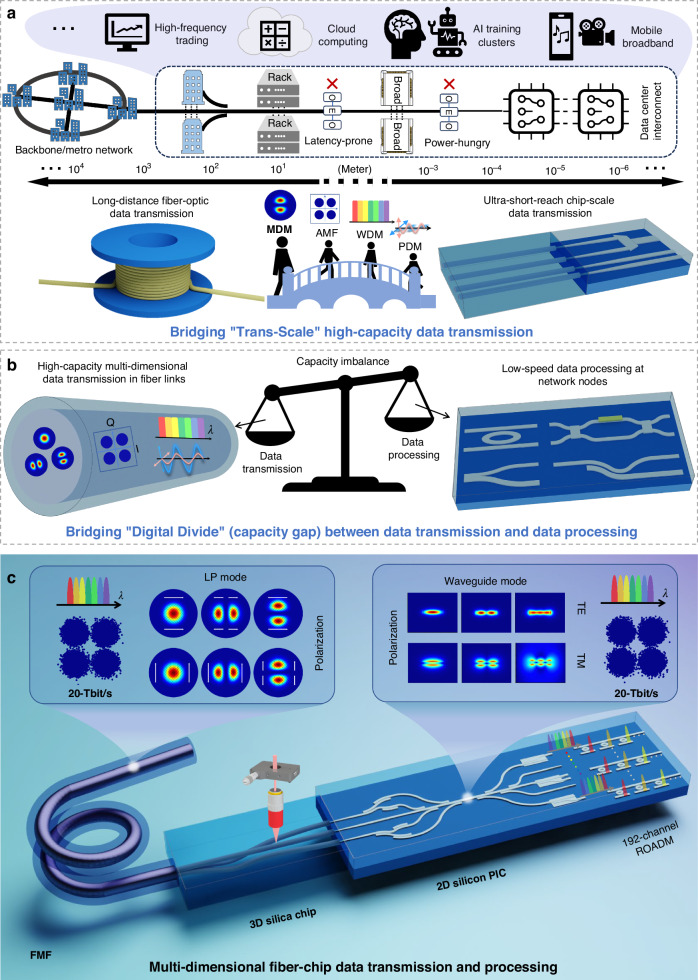
Trans-scale multi-dimensional fiber-chip data transmission and processing. **a** Artistic vision of high-capacity trans-scale bridging between long-distance fiber-optic data transmission and ultra-short-reach chip-scale data transmission. **b** Schematic of “Digital Divide” capacity gap between high-capacity data transmission in fiber links and low-speed data processing at network nodes. **c** Multi-dimensional fiber-chip data transmission and processing uses the FMF, 3D silica fs-laser direct writing photonic chip, and 2D silicon photonic integrated circuit (PIC) to realize 192-channel and 20-Tbit/s multi-dimensional (mode, polarization, wavelength) data transmission and processing. MDM mode-division multiplexing; PDM polarization-division multiplexing; WDM wavelength-division multiplexing; AMF advanced modulation format; FMF few-mode fiber; TE transverse electric; TM transverse magnetic

The emergence of big data and 5 G/6 G era has presented unprecedented challenges to the transmission and processing of massive data^1^, while also offering a new opportunity for optical communication technologies^2,3^. Hybrid multi-dimensional multiplexing technologies, which aim to explore and harness multiple physical dimensions of photons, have garnered significant attention to overcome the upcoming “capacity crunch” of single-mode fiber (SMF) based optical communications^4–9^. In recent years, the utilization of few-mode fiber (FMF) has enabled significant progress in multi-dimensional multiplexing, encompassing mode-division multiplexing (MDM), polarization-division multiplexing (PDM), wavelength-division multiplexing (WDM), and advanced modulation format (AMF) technology. The progress has facilitated the sustainable expansion of long-distance fiber-optic communication capacity for high-capacity data transmission in backbone and metro networks^10–14^. Meanwhile, driven by various dimensional integrated (de)multiplexers^15–18^, on-chip multimode waveguides (MMWs) have emerged as capable of facilitating ultra-short-reach high-throughput data transmission in data center interconnects (DCIs)^19^. In a communication system, fiber networks and MMW nodes serve as two terminal segments, each operating at a different scale. Traditional approaches to interconnect these terminals rely on multiple intermediate stages (rack, broad), additional interfaces, forwarding processes, and repeated optoelectronic conversions^20,21^. Each optical-electrical-optical (O-E-O) conversion introduces a latency-prone and power-hungry process. In the realm of future optical communications, seamless connection with high capacity between long-distance fibers and ultra-short-reach chips is one of the significant visions (see Fig. 1a). Such trans-scale transmission can eliminate redundant intermediate optoelectronic interfaces, enabling applications in high-frequency trading, cloud computing, AI training clusters, and mobile broadband. However, achieving this goal encounters a great challenge in high-capacity trans-scale bridging, primarily due to the lack of a scalable multi-dimensional fiber-chip interface, particularly concerning the spatial mode dimension.

In addition to data transmission, data processing is also of great importance in optical communications^22–24^. However, prevalent data processing technologies rely on discrete bulky devices characterized by high complexity, large volume, and high cost, which are not conducive to the miniaturization of processors^25,26^. Silicon photonics, an appealing integrated photonic platform, holds immense potential for chip-scale multi-dimensional data processing owing to its low power consumption, high-density integration, and complementary metal-oxide semiconductor (CMOS) compatibility^27–29^. Various silicon processors have demonstrated impressive performance^30–34^ and are rapidly advancing towards programmability^35–38^, reconfigurability^39–41^, multi-tasking^42,43^, and intelligence^44–47^. In this scenario, fiber-chip communication systems, capitalizing on optical fiber links for data transmission and integrated on-chip networks for data processing, are thus evolving into the mainstream architecture for transmitting and processing data in future modern communication networks^48–50^. Although silicon photonic processors achieve throughput exceeding 1 Tbit/s^51,52^, a significant “digital divide” still exists between high-capacity data transmission in fiber links and low-speed data processing at network nodes, impeding the rapid progress of optical communications (see Fig. 1b). In addition to the high-throughput chips needed to process multi-dimensional data, another major challenge associated with multi-dimensional fiber-chip systems is also the absence of aforementioned scalable multi-dimensional fiber-chip interface that ensures seamless high-capacity transmission between fibers and chips. In order to address the multi-dimensional fiber-chip interface challenge, researchers are actively seeking scalable solutions for the significant multimode mismatch in terms of shape and size between fiber-guided modes and chip-guided modes, such as linear polarization (LP) modes in FMFs and transverse electric/transverse magnetic (TE/TM) modes in multimode waveguides (MMWs)^8,53^. Traditional silicon multi-mode couplers, employed for connecting FMF and MMW, include various techniques such as vertical diffraction^48,50,54–59^, multi-mode conversion^53,60–62^, and power splitter and combiner^59,63–66^. However, they often exhibit compromised performance in terms of mode numbers, insertion loss, modal crosstalk, and fabrication tolerance (see Supplement 1, Section S1), thereby constraining the achievable capacity in scalable fiber-chip transmission and processing architectures.

In this paper, we propose and demonstrate a universal yet diverse strategy for realizing the high-capacity trans-scale bridging between long-distance fiber and ultra-short-reach chip by using the hybrid integrated coupler that consists of the 3D silica fs-laser direct writing photonic chip and 2D silicon photonic integrated circuit. By converting multi-mode coupling to the single-mode array coupling, the hybrid integrated coupler overcomes the multimode mismatch and implements the efficient multimode conversion of LP modes in FMF and TE/TM modes in MMW. In addition to the inherent advantages of hybrid integration, such as simplified design and enhanced compatibility^67^, our general strategy can be extended for the efficient coupling of various kinds of higher-capacity fibers (e.g. multi-mode fiber) and silicon MMWs due to the 3D processing capability of fs-laser direct writing technology^68^. Compared to previous work^69^, this hybrid integrated coupler implements the directly coupling between FMF and MMW without the need for additional single-mode fibers devices, featuring a more compact configuration. With these merits, as illustrated in Fig. 1c, we construct a multi-dimensional FMF-chip communication system enabling 192-channel and 20-Tbit/s trans-scale data transmission and processing, which represents, to the best of our knowledge, the largest number of data channels and highest data transmission and processing capacity ever the reported fiber-chip communications to date. In particular, a 2D silicon reconfigurable optical add/drop multiplexer (ROADM) with more than 2000 elements is employed for on-chip multi-dimensional data processing. In addition, due to the wide wavelength tuning range, each channel of the ROADM can add or drop arbitrary wavelength channels, enhancing the robustness of the presented multi-dimensional FMF-chip data transmission and processing system.

## Results

### Hybrid 2D/3D integrated multi-mode coupler

**Fig. 2:**
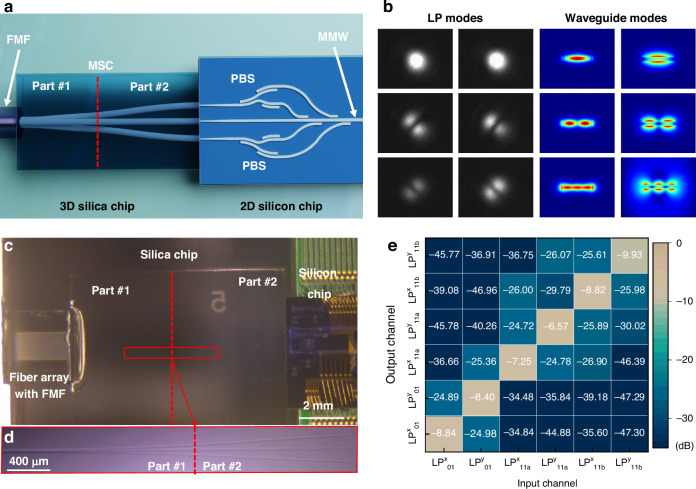
Hybrid 2D/3D integrated multi-mode coupling. **a** Concept of the hybrid 2D/3D integrated coupler consisting of 3D silica fs-laser direct writing photonic chip and 2D silicon photonic integrated circuit. **b** The measured intensity profiles of LP modes (LP^x/y^_01_, LP^x/y^_11a_, LP^x/y^_11b_) and simulated intensity profiles of waveguide modes (TE_0_, TM_0_, TE_1_, TM_1_, TE_2_, TM_2_). **c** Photograph of hybrid 2D/3D integrated multi-mode coupler and **d** the enlarged photograph of silica mode selective coupler. **e** Modal crosstalk matrix of hybrid 2D/3D multi-mode coupler at 1550 nm. MMW multi-mode waveguide; LP linear polarization; MSC mode selective coupler; PBS polarization beam splitter

Figure 2a illustrates the schematic of the hybrid 2D/3D integrated multi-mode coupler for trans-scale bridging between conventional FMF and silicon MMW. Hybrid integration fully capitalizes on the capabilities of both a 3D silica chip by fs-laser direct writing technology and a 2D silicon chip by lithography. The operational principle of the multimode coupler involves converting the multi-mode coupling into the single-mode array coupling. This approach not only maximizes the benefits of the mature single-mode coupling technology but addresses the challenge of significant mode mismatch between LP modes and waveguide modes, as shown in Fig. 2b. The coupling process from LP modes in FMF to waveguide modes in MMW is as follows. Initially, multiple LP modes are coupled into the silica multimode waveguide via the edge coupling method and subsequently separated into an array of single-mode waveguides using a silica mode selective coupler (MSC). Each single-mode waveguide supports two orthogonal polarizations. Then, a linear array of inverse tapers facilitates a low-loss connection between the silicon chip and the silica chip. Finally, the dual-polarized fundamental modes of three silicon waveguides are multiplexed into the high-order modes in silicon waveguides through the polarization beam splitters (PBSs) and asymmetric directional coupler (ADC) based mode multiplexers. Following the reciprocity law of light, high-order waveguide modes can be converted into LP modes and coupled back to the FMF through a reverse process. A photograph of the hybrid 2D/3D integrated multi-mode coupler is depicted in Fig. 2c. For further details on the manufacturing processes of the 3D silica and 2D silicon chips, please refer to Supplement 1, Sections S2 and S3.

Figure 2d presents an enlarged photograph of silica MSC, featuring three single-mode ports, a multimode port, and three approaching waveguides. The single-mode port and multimode port boast diameters of 9 μm and 14 μm, respectively, facilitating low-loss propagation of the fundamental mode and high-order mode. The spacing between single-mode waveguide ports is set to a fixed distance of 127 μm, ensuring compatibility with a commercial fiber array for performance measurement and system experimentation. The MSC comprises two main parts. Part #1, connected to the multimode port, adjusts the coupling parameters (waveguide spacing and coupling length) of three approaching waveguides to convert and redistribute the light, enabling the mapping of three different LP modes onto a triangle single-mode array (see Supplement 1, Section S4). The triangular array has a side length of 40 μm, preventing coupling between adjacent waveguides. Part #2 of the MSC employs 3D trajectories to transition the waveguide distribution from a triangular array to a linear one, facilitating the hybrid integration of 2D and 3D photonic chips. Utilizing mature single-mode edge coupling technology, three 160-μm-long tapered waveguides establish connections between the silicon chip and silica chip with the insertion loss of 2 dB and polarization-dependent loss of 0.5 dB (see Supplement 1, Section S5). Performance enhancement is achievable through specialized structures such as multi-tip tapers^70^, SWG waveguides^71,72^, et al. Due to the polarization dependence of ADC-based mode multiplexers, dual polarizations (TM_0_ mode and TE_0_ mode) in single-mode silicon waveguides should be separated into two channels using a fabrication-tolerant silicon PBS. The silicon PBS incorporates an improved structure with cascaded bent directional couplers^73^ to achieve high performance including an insertion loss of < 0.5 dB and crosstalk of <-35 dB covering the C band (see Supplement 1, Section S6). Subsequently, the on-chip high-order modes can be multiplexed and obtained by the cascaded ADC structures. Previous analyses^74,75^ have indicated that the sidewall error in fabricated waveguides can degrade the performance of two-taper ADC mode multiplexers due to mode hybridization. Therefore, to ensure high performance and fabrication tolerance, the adiabatic coupling region of ADC structures should be carefully designed to avoid any regions prone to mode hybridization. If there is a partial overlap between the width range of the coupling region and the hybridization region, ADC structures with shorter coupling lengths should be considered as potential solutions. Further details regarding the design and simulated results are provided in Supplement 1, Section S7, which demonstrates an insertion loss of <1 dB and crosstalk of <-30 dB covering the C band for the TM_2_, TE_2_, TM_1_, and TE_1_ modes.

The total insertion loss of the hybrid 2D/3D integrated coupler is measured to be approximately 5 dB, including the MSC loss of 1.5 dB, single-mode coupling loss of 2 dB, PBS loss of 0.5 dB, and ADC-based multiplexers of 1 dB. In addition to the insertion loss, the inter-modal crosstalk is crucial for assessing the performance of the hybrid integrated multimode coupler. Detailed measurements are provided in Supplement 1, Section S8. As depicted in Fig. 2e, the inter-modal crosstalk among the six modes registers below −16 dB at a wavelength of 1550 nm. Further results across various wavelengths are presented in Supplement 1, Section S8, indicating that inter-modal crosstalk remains consistent below −15 dB across all measured wavelengths, similar to the values obtained at 1550 nm.

### Large-scale 2D silicon ROADM

**Fig. 3:**
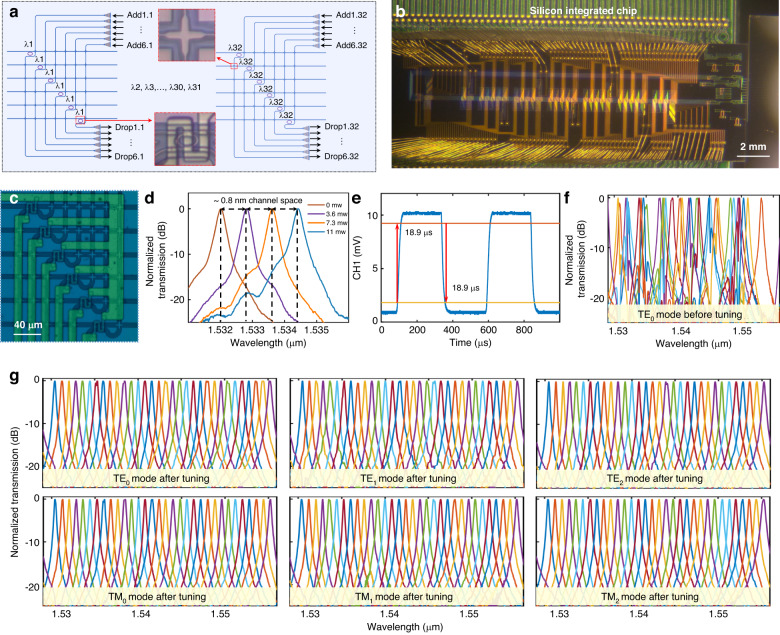
Large-scale 2D silicon ROADM. **a** Configuration of silicon ROADM consisting of microring-resonator array with more than 2000 elements. The insets are the microscopy images of fabricated silicon crossing and microring-resonator. **b** The microscopy images of the fabricated silicon ROADM. **c** The enlarged view of the MRR-based array. **d** Measured transmissions and **e** switching responses of the microring resonator with different heating voltages. **f** The measured drop spectra of the array of 32 cascaded microring-resonators without thermo-optical tuning (@TE_0_ mode). **g** The measured drop spectra of six arrays of 32 cascaded microring-resonators with thermo-optical tuning

As illustrated in Fig. 3a, the schematic of the 2D silicon ROADM consists of six arrays of 32 cascaded wavelength-selective microring resonators (MRRs), serving the data processing function. Figure 3b and 3c display the microscopy images of the fabricated silicon ROADM and the enlarged view of the MRR-based array, respectively. Notably, the large-scale silicon photonic chip integrates monolithically over 2000 elements, including 1152 waveguide crossings, 384 single-mode grating couplers, 192 MRRs, 192 micro-heaters, and 224 pads. Employing the particle swarm optimization method, the waveguide crossing achieves ultra-low loss below 10 mdB, essential for reducing loss and simplifying layout in advanced large-scale photonic systems^76^. A shallow-etched grating coupler with a small footprint provides a single-mode interface for adding and dropping functions. Each MRR serves as a key WDM element using its inherent resonance characteristics. As depicted in the inset of Fig. 3a, the wavelength-selective switch utilizes an elliptical microring with adiabatically varied radius and core width. As demonstrated in our previous work^77,78^, the relatively large bending radius and narrow waveguide width are designed in the coupling region to obtain sufficient coupling efficiency, while the relatively small bending radius and broadened core width are employed to reduce the bending loss and the cavity length. The reduced cavity length contributes to achieving a broad free spectral range (FSR) of ~28 nm, supporting 32 wavelengths with a channel spacing of ~0.8 nm (~100 G). Unlike traditional resonant MRR aiming for critical coupling, the present MRR slightly shortens the coupling waveguide spacing to increase the coupling efficiency, achieving a state of “under coupling”. In the state of “under coupling”, the 3-dB bandwidth of the resonant peak can be broadened with the slight sacrifice of the insertion loss, facilitating higher-rate signals. Figure 3d displays the measured transmission spectra of the MRR-based wavelength selective switch under different heating voltages. Voltage variations of 0.72 V, 1.14 V, and 1.5 V induce red shifts of approximately 0.8 nm, 1.6 nm, and 2.4 nm in the resonant peak, supporting wavelength-selective adding/dropping. Moreover, one can see that the 3-dB bandwidth is approximately 0.2 nm and the average 0.8 nm inter-wavelength crosstalk is less than −15 dB. In Fig. 3e, the MRR-based wavelength selective switch exhibits a switching rise-time and drop-time of 18.9 μs each, enabling rapid wavelength switching with 20 μs. Although fabrication errors may cause variations in wavelength shifts among the 32 cascaded MRRs (see Fig. 3f), thermo-optic tuning ensures uniform channel spacing and minimal inter-wavelength crosstalk. Detailed information about MRR thermal crosstalk can be discussed in Supplement 1, Section S9. Figure 3g illustrates the normalized drop spectra of the 6 arrays of 32 cascaded MRRs with thermal tuning, corresponding to the TE_0_, TE_1_, TE_2_, TM_0_, TM_1_, and TM_2_ mode channels. The average inter-wavelength crosstalk remains −15 dB. The uniform distribution of 32 resonant wavelengths across the six mode channels confirms the successful implementation of the 192-channel ROADM within the 2D silicon photonic integrated circuit.

### Trans-scale multi-dimensional FMF-chip data transmission and processing

**Fig. 4:**
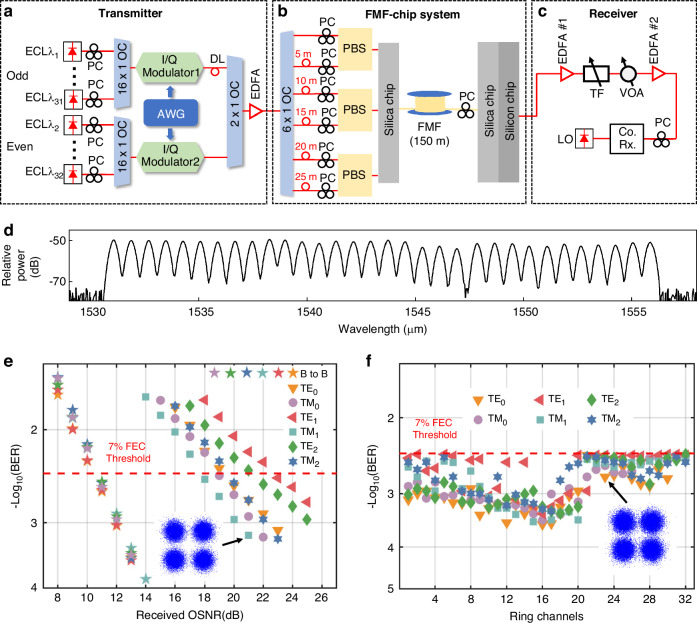
Multi-dimensional FMF-chip communication. The experimental setup for **a** the transmitter, **b** the FMF-chip system, and **c** the receiver. **d** An optical spectrum of 32-wavelength WDM signals with ~ 100 G channels spacing (from 1530.4 nm to 1555.2 nm). **e** Measured BER vs. OSNR curves for six modes of different wavelengths. **f** The measured BERs of all 192 channels after FMF-chip system. The inserts in **e** and **f** are the typical constellations of QPSK signals. ECL External cavity laser; DL delay line; PC Polarization controller; OC Optical coupler; AWG Arbitrary waveform generator; EDFA Erbium-doped fiber amplifier; VOA Variable optical attenuator; TF tunable filter; Co.Rx. coherent receiver; LO local oscillator

In this section, we utilize the hybrid 2D/3D integrated multi-mode coupler and large-scale ROADM to construct an FMF-chip system, enabling 192-channel and 20-Tbit/s trans-scale multi-dimensional data transmission and processing. Figure 4a–c illustrate the entire experimental setup for multi-dimensional data transmission and processing, which mainly consists of three parts: the WDM signal transmitter with quadrature phase-shift keying (QPSK) modulation, a hybrid integrated FMF-chip system, and the coherent optical receiver followed by digital signal processing (DSP). At the transmitter, 32 wavelength-tunable external cavity lasers (ECLs) serve as optical signal carriers with wavelengths spaced at a 0.8-nm/100-GHz grid. To emulate real communication scenarios, we divide the 32 ECLs into odd and even groups and independently modulate them with two 56-GBaud QPSK signals using a four-channel arbitrary waveform generator (AWG: Keysight M8199A) to drive two in-phase/quadrature (I/Q) modulators. An optical delay line can be used to decorrelate the odd and even optical signals, thereby avoiding underestimation of the bit-error ratio (BER) in WDM systems. The modulated odd and even signals are combined via a 50:50 optical coupler (OC) and amplified by a C-band erbium-doped fiber amplifier (EDFA). Subsequently, the WDM signal with 32 channels from 1530.4 nm to 1555.2 nm is transmitted to the FMF-chip data transmission and signal processing system, as illustrated in Fig. 4d. It can be seen that the wavelength channel spacing is not very strictly uniform (wavelength shift of < 0.1 nm) due to the limitation in the accuracy of laser wavelength tuning, leading to slight fluctuation in inter-wavelength crosstalk and BER. The amplified signal is split into 6 copies and further decorrelated using different optical delay lines. These six channels are multiplexed into six different LP modes in FMF using three fiber-based PBSs and the silica MSC. Following the transmission in FMF, multi-dimensional signals including 3 modes, 2 polarizations, and 32 wavelengths, are coupled into the silicon MMW by the hybrid integrated coupler. Then, the cascaded mode and polarization handling devices including ADC-based demultiplexer, polarization splitter rotation, and polarization rotation (see Supplement 1, Section S6) are employed for demultiplexing from six modes in MMW to six TE_0_ modes in single-mode waveguides, owing to the TE fundamental mode operation of the silicon ROADM. Consequently, the 192-channel signals can be flexibly dropped from the 2D photonic integrated circuit and sent to the integrated coherent optical receiver and local oscillator (LO). A real-time oscilloscope (Keysight UXR0594) is utilized to sample and store the electrical signals from the coherent optical receiver for offline DSP and BER evaluation. The offline DSP mainly involves signal resample, any IQ non-orthogonality compensation with Gram Schmidt orthogonalization procedure, linear equalization, and carrier recovery.

**Fig. 5:**
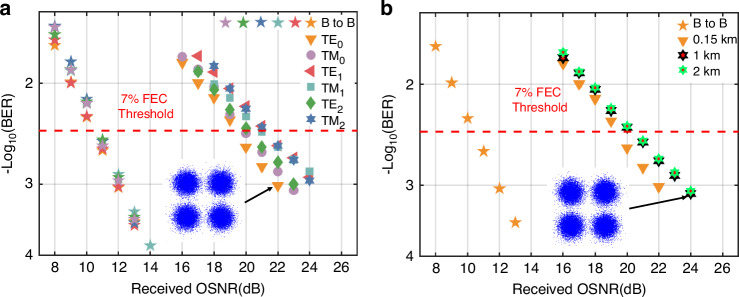
The measured BER curves for the adding function. **a** six mode/polarization/wavelength channels. **b** FMF lengths are 0.15 km, 1 km, and 2 km

Figure 4e depicts the measured BER plotted against the received optical signal-to-noise ratio (OSNR) when dropping six mode channels of different wavelengths from the silicon ROADM. For the sake of comprehensiveness and simplification, six mode channels evenly select the 1st (@TM_0_ mode), 6th (@TM_2_ mode), 13th (@TE_0_ mode), 19th (@TE_1_ mode), 27th (@TE_2_ mode), 32nd (@TM_1_ mode) wavelength channels. One can see that the BER curves of six selected channels consistently fall below the 7% forward error correction (FEC) threshold of 3.8 × 10^−3^, despite inter-modal crosstalk and inter-wavelength crosstalk, when OSNRs exceed 23 dB. Comparing the BER performance with the back-to-back transmission of each channel, the observed OSNR penalties of six selected channels in the FMF-chip system are less than 13 dB at a BER of 7% FEC threshold. In addition, Fig. 4e shows a 6 dB OSNR penalty gap between different modes, resulting from fluctuating insertion loss and intermodal crosstalk. This gap can be reduced by either improving fabrication precision or incorporating an optical power equalizer to compensate for mode-dependent variations. Regarding the adding function, the observed OSNR penalties of six mode channels remain below 11 dB, demonstrating similar performance to the dropping function (see Fig. 5a). The chosen mode/polarization/wavelength channels are the same as the dropping function in Fig. 4e, to give a performance comparison. For the 7% soft-decision FEC threshold of 3.8 × 10^−3^, the OSNR penalty values are around 9–11 dB for six selective channels. The measured BERs can be below the 7% soft-decision FEC threshold of 3.8 × 10^−3^ with inter-wavelength crosstalk and inter-mode crosstalk when OSNR is over 23 dB, which is similar to that of the dropping function. Additionally, Fig. 5b presents the BER curves for different FMF lengths. Due to the limitations of available FMF in the laboratory, 2 km is the maximum fiber length used in the experiment. As shown in the BER results, increasing the FMF length from 0.15 km to 1 km introduces an OSNR penalty of approximately 1 dB. However, extending the fiber from 1 km to 2 km results in only a slight additional OSNR penalty of about 0.1 dB. This result suggests that our system can potentially support longer FMF transmission.

To showcase the data throughput, each mode/polarization/wavelength channel in the hybrid integrated FMF-chip system undergoes asynchronous demultiplexing processing, reception, and testing individually. Figure 4f shows the measured BER values of all 192 channels in the multi-dimensional data transmission and processing scenario, showing that all BER values remain below the 7% FEC threshold of 3.8 × 10^−3^. The typical constellations of QPSK signals in insets highlight the favorable performance. Therefore, the multi-dimensional data transmission and signal processing system successfully achieves an aggregate capacity of 20.01 Tbit/s (56 GBaud × 2 bits per symbol × 3 modes × 2 polarizations × 32 wavelengths ⁄ (1 + 7%) ≈ 20.01 Tbit/s).

### Robustness demonstration

**Fig. 6:**
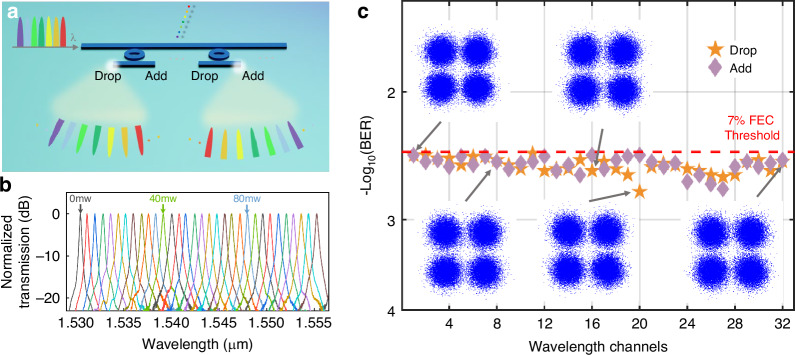
Robustness demonstration. **a** The scheme of the MRR with large tuning wavelength range. **b** Normalized transmission spectra at the drop port of the single MRR-based wavelength switch (@ TE_0_ mode) as the applied heating power increases from 0 to 113 mW. It can be clearly observed that the resonant wavelength is red-shift with a step of 0.8 nm. **c** The BER values of 32 wavelengths from single MRR-based wavelength switch when the signals are dropped and added from the FMF-chip system. The insets in **c** are the typical constellations of QPSK signals

In scenarios where the multichannel system incorporating a large-scale silicon photonic integrated circuit encounters operational challenges such as fabrication errors or incorrect operation, it may result in the disabling of one or more channels, thereby restricting its practical utility in communication applications. Remarkably, our multi-dimensional data transmission and processing system offers a solution to mitigate such limitations. For the ROADM composed of two AWGs and an MZI array, each wavelength is strictly routed through a fixed port^69^. Damage to any single port will result in the permanent failure of its corresponding wavelength channel. As illustrated in Fig. 6a, the utilization of a single MRR-based switch in the presented system enables flexible processing of arbitrary wavelengths, owing to its wide wavelength tuning range spanning more than an FSR. Figure 6b depicts the normalized transmission spectra at the drop port as the applied heating power increases, showcasing a clear red shift of the resonant wavelength with a step of 0.8 nm. Furthermore, Fig. 6c presents the BER values obtained from 32 wavelengths when employing a single MRR-based wavelength switch for dropping and adding 56-GBaud QPSK signals in the FMF-chip system. The BER values, all below the 7% FEC threshold, affirm that processing arbitrary wavelengths using a single MRR does not compromise the system capacity. If one port is damaged, there are still 31 remaining ports capable of handling the data associated with that wavelength. These 31 ports, if otherwise idle, can be reassigned to process the data of that specific wavelength. Therefore, the failure of a single port does not render the corresponding wavelength unusable, thereby significantly enhancing the robustness of the multi-dimensional FMF-chip data transmission and processing system.

## Discussion

In summary, we bridge “Trans-Scale” high-capacity data transmission between long-distance fiber and ultra-short-reach chip and “Digital Divide” between high-capacity data transmission in fiber links and low-speed data processing at network nodes. The key device is the diverse hybrid 2D/3D integrated multimode coupler consisting of the 2D silicon photonic integrated circuit and 3D silica fs-laser direct writing photonic chip, which effectively addresses the significant mode mismatch between the LP modes and waveguide modes and bridges trans-scale data transmission between FMF and silicon MMW. Combined with a large-scale silicon ROADM, a high-capacity and multichannel FMF-chip system is constructed to facilitate trans-scale multi-dimensional data transmission and processing. Notably, the monolithic integration of the large-scale silicon ROADM encompasses over 2000 elements, enabling the flexible processing of 192-channel mode/polarization/wavelength signals. It is worth mentioning that the large-scale silicon chip in the system possesses a greater number of channels and a higher throughput compared with all reported silicon integrated chips (see Supplement 1, Section S10)^15,17,18,41,79–81^. By carrying 56-Gbaud QPSK signals, our multi-dimensional FMF-chip system achieves a groundbreaking 20-Tbit/s data transmission and processing capability. With the goal of practicality, based on the broad wavelength tuning range, the MRR-based switch can process the arbitrary wavelength, which relaxes the limitation of disability for some channels, thereby enhancing the robustness of the data processing system. We believe that our demonstration is expected to provide a trans-scale architecture for multi-dimensional data transmission and processing in next-generation optical communications.

**Fig. 7:**
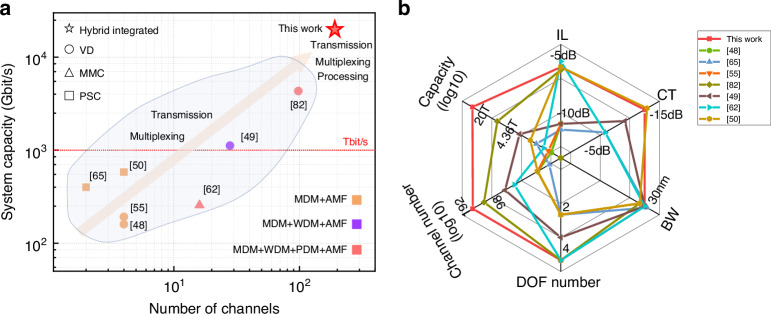
Comparison of reported fiber-chip communications. **a** The development trend of fiber-chip communication in terms of channel number and system capacity. **b** Radar charts of fiber-chip communications. Six coordinates include the performance of multi-dimensional interface (insertion loss (IL), crosstalk (CT), and bandwidth (BW)) as well as performance of fiber-chip system (DOF number, channel number, and capacity). MDM mode-division multiplexing; PDM polarization-division multiplexing; WDM wavelength-division multiplexing; VD vertical diffraction; MMC multi-mode conversion; PSC power splitter and combiner

Figure 7 shows a comprehensive overview of the current state-of-the-art FMF-chip systems (more details can be found in Supplement 1, Section S10). In Fig. 7a, we highlight the progress made in this work in terms of their system capacity and the channel number. Our demonstration is the first one that simultaneously enables 192-channel and 20-Tbit/s capacity for trans-scale data transmission and processing, compared with these reported systems that exhibit fewer channels, fewer multiplexing dimensions, and lower system capacity. Moreover, it becomes evident that most of reported FMF-chip systems perform the function of data transmission and (de)multiplexing but do not have data processing capabilities. In ref. ^82^, slightly efficient coupling of four polarization/modes can be realized by the 2D grating. Despite compactness, this structure can enable a 98-channel chip-fiber-chip system with an only capacity of 4.36 Tbit/s. Furthermore, a rectangular core FMF with the mode fields regularly distributed along one transverse direction is proposed to achieve the four-mode coupling between fiber and waveguide by inverse design grating^49^. For the specially designed FMF, more complex fiber manufacturing technology and the incompatibility with modern fiber links both are detrimental to the scalability of FMF-based transmission and processing links toward higher capacity. In contrast, our hybrid 2D/3D integrated coupler exhibits a broader application prospect owing to the compatibility with communication systems of conventional FMF. In Fig. 7b, the radar chart visualizes the performance of multi-dimensional interface (insertion loss, crosstalk, and bandwidth) as well as performance of fiber-chip system (degrees of freedom (DOF) number, channel number, and capacity). It can be clearly seen that all performance of our multi-dimensional interface is at the advanced level as the reported optimal performance of coupler, which contributes to a record-large channel number and a record-high capacity of fiber-chip communications. Specifically, our coupler has significant advantages in scalability due to the diversity of 3D chips. Overall, we believe that our work represents a significant step towards achieving twenty-terabit-per-second data transmission and processing, highlighting the potential of combining optical fiber links and photonic integrated chips for future high-capacity and highly integrated optical communication systems.

Currently, the conventional FMF is used in our trans-scale multi-dimensional fiber-chip system. Benefiting from the 3D processing capability of fs-laser direct writing technology, we can directly connect and couple various kinds space-division multiplexing (SDM) fibers such as single-mode multicore fiber, few-mode multicore fiber^83^, and orbital angular momentum (OAM) fiber^84^ into the silicon MMW. This advancement enables the realization of diverse trans-scale fiber-chip communication systems and further enhances the system capacity and scalability. Cascaded MRRs^77,78^ widen the 3-dB bandwidth, enable the transmission of higher data rate signals, and enhances system capacity. MRR with an FSR of up to 93 nm has already been reported^85^, indicating the potential for further increasing the number of wavelength channels. However, due to the cascaded architecture, the insertion loss of the devices should be carefully considered when scaling up the number of wavelengths. To reduce the insertion loss of the hybrid 2D/3D integrated coupler, we can employ more efficient single-mode edge couplers, such as foundry-compatible bi-level waveguides^86^, as well as advanced silicon waveguide structures^67,70^. Furthermore, the fiber-chip system can incorporate more complex functionalities. For instance, the integration of a non-block switch^32^ placed before the multichannel ROADM facilitates the processing of arbitrary channel signals by arbitrary MRRs within the entire system. An intelligent optical processor can be integrated to reduce crosstalk and perform an all-optical MIMO descrambler^58^. It is noteworthy that the response speed of MRR can range from around 20 µs when using thermo-optic phase shifters to less than a nanosecond when employing electro-optic phase shifters^87^. This capability holds the potential for real-time data processing in multi-dimensional fiber-chip communication systems.

## Materials and methods

### Fabrications

The 2D silicon photonic integrated circuit is fabricated using the standard CMOS-compatible fabrication process, including four-step electron beam lithography (EBL), three-step inductively coupled plasma (ICP) etching, three-step electron-beam evaporation (EBE), three-step deep ultra-violet (DUV) lithography, two-step plasma-enhanced chemical vapor deposition (PECVD), and one-step reaction ion etching (RIE). The first EBL step and first EBE step are used to form the Au marks on a silicon-on-insulator (SOI) wafer for alignment. The three EBL steps assisted with three ICP steps are employed to define the 70-nm-etch, 150-nm-etch, and 220-nm-etch waveguide patterns and transfer them onto the SOI wafer, respectively. Then, a 1.5-μm-thick SiO_2_ cladding layer covering the entire device is deposited by the first-step PECVD. After that, a Ti layer for heating and an Au layer for the electrode pad both are formed by DUV lithography, EBE, and the lift-off process. Finally, a thin SiO_2_ cladding layer is deposited by the second PECVD step to protect the heater and electrode pad and the last DUV step and RIE are used to remove the dielectric stack on the pad. More fabrication details about silicon chips are given in Supplementary 1, section S2.

The 3D silica chip is processed by the fs-laser fabrication technology with a high repetition rate Ti: Sapphire oscillator (1030 nm wavelength, 200 kHz repetition rate, 234 fs pulse duration). The linear polarization fs-laser is vertically focused ~50 μ*m* below the top surface of a silica photonic chip through a 50 × 0.42 objective. The laser beam profile is modified by a linear slit, which is used to inscribe low-loss circular waveguides in glass. In addition, the fabricated waveguides with twice inscribed traces are proposed to improve the refractive index and ensure the smoothness of the waveguide. The inscribed speed of fs-laser is a constant of 0.2 mm/s. The refractive index contrast of fabricated waveguides is approximately 0.3%. More fabrication details about femtosecond laser fabrication technology can be found in Supplementary 1, section S3.

### Measurements

The polarization/mode multiplexer and cascaded ADC-based mode demultiplexer are placed on the two sides of the hybrid 2D/3D integrated coupler for the crosstalk measurement. The polarization/mode multiplexer consisting of a silica MSC and three fiber-based PBS is used to obtain three LP modes with dual polarizations in FMF. Six waveguide modes are monitored and demultiplexed into six TE_0_ modes by the cascaded ADC-based mode demultiplexer and silicon PSR. A tunable laser (Santec TSL-710) provides the light covering the C band and its polarization is adjusted by a polarization controller. Finally, the light is monitored by the optical power meter (PMSII-A). More details on the measurement of the 2D/3D integrated coupler can be found in Supplementary 1 section S8.

## Supplementary information

Supplementary_Material

## Data Availability

All data are available in the main text or the supplementary materials.
