# Supplementary material for: Harnessing diverse hybrid integration for bridging trans-scale multi-dimensional fiber-chip data transmission and processing
Source: Light Sci Appl. 2026 Mar 12;15:167. doi: 10.1038/s41377-026-02194-9 (PMC12982771; doi:10.1038/s41377-026-02194-9)
Supplement: Supplementary file 1 — Supplementary_Material [file 41377_2026_2194_MOESM1_ESM.docx]

**Supplementary material for**

**“Harnessing diverse hybrid integration for bridging trans-scale multi-dimensional fiber-chip data transmission and processing”**

*Kang Li^1,2,3^, Guofeng Yan^1,2,3^, Kangrui Wang^1,2,3^, Chengkun Cai^1,2,3^, Min Yang^1,2,3^, Guangze Wu^1,2,3^, Weike Zhao^4^, Yingying Peng^4^, Yaocheng Shi^4^, Daoxin Dai^4^, and Jian Wang^1,2,3*^*

*^1^ Wuhan National Laboratory for Optoelectronics and School of Optical and Electronic Information, Huazhong University of Science and Technology, Wuhan 430074, Hubei, China*

*^2^ Hubei Optical Fundamental Research Center, Wuhan 430074, Hubei, China*

*^3^ Optics Valley Laboratory, Hubei, Wuhan 430074, Hubei, China*

*^4^ State Key Laboratory for Modern Optical Instrumentation, Center for Optical & Electromagnetic Research, College of Optical Science and Engineering, International Research Center for Advanced Photonics, Zhejiang University, Zijingang Campus, Hangzhou 310058, China*

** Corresponding author:* [*jwang@hust.edu.cn*](mailto:jwang@hust.edu.cn)

**Contents**

[**S1: Comparisons of the reported silicon multimode fiber-chip coupler 1**](#_Toc215746732)

[**S2: Silicon process technology 3**](#_Toc215746733)

[**S3: Femtosecond laser direct writing technique 5**](#_Toc215746734)

[**S4: 3D silica device 6**](#_Toc215746735)

[**S5: Single-mode and multi-mode coupling of the presented hybrid coupler 10**](#_Toc215746736)

[**S6: Silicon polarization handing devices 12**](#_Toc215746737)

[**S7: Silicon mode multiplexers 16**](#_Toc215746738)

[**S8: Measurement of the inter-modal crosstalk for the multi-mode coupler 19**](#_Toc215746739)

[**S9: MRR thermal crosstalk 21**](#_Toc215746740)

[**S10: Comparisons of different kinds of integrated chip and fiber-chip systems 22**](#_Toc215746741)

[**References 23**](#_Toc215746742)

# S1: Comparisons of the reported silicon multi-mode fiber-chip coupler

**Table S1** Comparison of the reported silicon multi-mode fiber-chip coupler

| Principles | Fiber modes | Waveguide modes | Insertion loss (dB) | Crosstalk (dB) | Wavelength (nm) | Structures | EXP or SIM | Ref. (year) |
| --- | --- | --- | --- | --- | --- | --- | --- | --- |
| VD | LP_01_, LP_11a_ | TE_0_, TE_1_ | < 18 | < -5.25 | 1550 | 1D grating | EXP | [54]  2018 |
| VD | LP_01_, LP_11a_,  LP_11b_ | TE_0_ array | < 9 | -- | 1570 | 2D grating, 4×4 MMI | EXP | [55]  2020 |
| VD | LP_01_^x/y^, LP_11_^x/y^ | TE_0_, TE_1_ in two input ports | < 9.1 | -- | 1542.1 - 1557.1 | 2D grating | EXP | [48]  2020 |
| VD | LP_01_, LP_11_ | TE_0_, TE_1_ | < 8.3 | -- | ~ 1530 | Mikaelian lens, 1D grating | EXP | [56] 2021 |
| VD | LP_01_, LP_11_ | TE_0_, TE_1_ | < 3.21 | -- | 1524 - 1556 | Two-layer grating | EXP | [57] 2022 |
| VD, PSC | LP_11_ | TE_1_ | < 5.68 | < -23 | 1533 - 1567 | ADC, 1D grating | EXP | [59] 2023 |
| MMC | LP_01_^x/y^, LP_11a_^x/y^, LP_11b_^x/y^ | TE_0_, TE_1_, TE_2_, TM_0_, TM_1_, TE_3_ | < 0.2 | < -23 | 1550 | Multistage taper, SiN waveguide | SIM | [53] 2015 |
| MMC | LP_01_^x/y^, LP_11a_^x/y^, LP_11b_^x/y^ | TE_0_, TE_1_, TE_2_, TM_0_, TM_1_, TM_2_ | < 0.6 | < -25 | 1550 | Multistage taper, tapered fiber | SIM | [60] 2022 |
| MMC | LP_01_^x/y^, LP_11a_^x/y^, LP_11b_^x/y^ | TE_0_, TE_1_, TE_2_, TM_0_, TM_1_, TM_2_ | < 0.6 | < -19 | 1530 - 1565 | Multistage taper, tapered fiber | SIM | [61] 2022 |
| MMC | LP_01_^x/y^, LP_11a_^y^, LP_11b_^y^ | TE_0_, TE_1_, TE_2_, TM_0_ | < 4 | < - 6.8 | 1570 - 1582 | 3D polymer taper | EXP | [62] 2023 |
| VD, PSC | LP_01_^x/y^, LP_11a_^x/y^, LP_11b_^x/y^ | TE_0_ array | < 23 | -- | ~ 1540 | 2D grating, 1×4 MMI | EXP | [63] 2013 |
| PSC | LP_01_, LP_11a_ | TE_0_, TE_1_ | < 7.39 | -- | 1515 - 1585 | Y-junction | EXP | [64] 2017 |
| PSC | LP_01_^x/y^, LP_11a_^x/y^, LP_11b_^x/y^, | TE_0_, TE_1_, TM_0_, TM_1_ | < 10.77 | < -10 | 1520 - 1610 | 1×3 MMI, triple-tip taper | EXP | [65] 2020 |
| PSC | LP_01_^x/y^, LP_11a_^x/y^ | TE_0_, TE_1_, TM_0_, TM_1_ | < 5.1 | < -25 | 1450 - 1650 | ADC, triple-tip taper | SIM | [66] 2022 |
| **HI** | **LP_01_^x/y^, LP_11a_^x/y^, LP_11b_^x/y^,** | **TE_0_, TE_1_, TE_2_, TM_0_, TM_1_, TM_2_** | **< 5** | **< -15** | **1530 - 1565** | **3D chip, 2D chip** | **EXP** | **This work** |

VD: Vertical diffraction; MMC: Multi-mode conversion; PSC: Power splitter and combiner; HI: Hybrid integration. EXP: experiment; SIM: simulation; ADC: asymmetric directional coupler.

The associated reference labels are from the Ref. [48, 53-57, 59-66] of the main text.

Table S1 summarizes the reported silicon multi-mode fiber-chip couplers for few-mode fiber (FMF) in terms of principle, mode number, insertion loss, crosstalk, wavelength, and structure. Specifically, although some multi-mode chip-fiber interfaces have been implemented with vertical grating structures, the diffractive coupling method faces inherent shortcomings such as bandwidth limitation and low diffraction efficiency^54-56^. Multi-mode communication systems employing 2D gratings may also require a reconfigurable photonic processor for performing multiple-input multiple-output (MIMO) functions^57-59^. While the multi-mode conversion method can achieve effective coupling, it entails a complex and slow evolution process and relies on a sufficiently thick low-index auxiliary waveguide, both of which pose higher demands on fabrication and hinder high-volume integration^60,61^. Furthermore, the high degeneracy of high-order modes significantly increases the difficulty of mode conversion^62^. Regarding the power splitter and combiner, the combined use of multi-mode interference (MMI) and triple-tip inverse tapers has enabled efficient four-mode coupling between not only traditional circular FMF and silicon MMW^64^ but also specific rectangular FMF and silicon MMW^50^. However, this approach is suitable for some high-order LP modes whose field profiles have multiple peaks in the horizontal direction, hindering further scaling of fiber-chip system capacity. Consequently, multi-mode couplers reported so far face challenges in scalability, impeding the development of higher-capacity fiber-chip transmission and processing systems.

# S2: Silicon process technology

In this section, the 2D silicon photonics chip is processed on a standard silicon-on-insulator (SOI) wafer with a 220 nm-thick top silicon layer and a 2-μm-thick buried oxide (SiO_2_) layer by using a combination of electron beam lithography (EBL) and deep ultraviolet (DUV) lithography, as shown in Fig. S1.

Firstly, four EBL steps (from Fig. S1**a** to **d**, from **e** to **h**, from **i** to **l**, and from **m** to **p**) are employed to fabricate the high-precision micro/nanostructure. Before each EBL step, ultrasonic baths in acetone, isopropyl alcohol, and DI water are successively utilized to ensure the cleanliness of the SOI wafer. After drying under nitrogen flow, the positive photoresist is spin-coated onto the SOI wafer surface and dried to remove moisture. The patterns can be transferred to the photoresist by the processes of EBL, development, and fixation. The alignment marks consisting of 50-nm-thick gold (Au) with 10-nm-thick chromium (Cr) are fabricated by the processes of first-step EBL, electron-beam evaporation (EBE), and lift-off. The three EBL steps assisted by three-step inductively coupled plasma (ICP) etching define the 70-nm-etch, 150-nm-etch, and 220-nm-etch waveguide patterns and transfer them onto the SOI wafer, respectively. Subsequently, a 1.5-μm-thick SiO_2_ cladding layer covering the entire device is deposited by plasma-enhanced chemical vapor deposition (PECVD).

Then, three DUV lithography steps (from Fig. S1**r** to **t**, from **u** to **w**, and from **x** to **y**) are employed to fabricate the heating phase shifts and electrode pads. Similar to the above-mentioned EBL process, the negative photoresist for DUV lithography is spin-coated onto the SOI wafer surface. The chromium and titanium layers are successively deposited by EBE, and the final heating phase shifts are formed by the lift-off process. The same DUV lithography is used to fabricate the electrode pads including the Cr and Au layers, where part of the electrode pads is deposited directly on the heating phase shifts for conducting electricity. Finally, a thin SiO_2_ cladding layer is deposited by the second-step PECVD to protect the heater phase shifts and electrode pads and the last DUV step and reactive ion etching (RIE) are used to remove the dielectric stack on the pad, providing a port for the applied voltage.


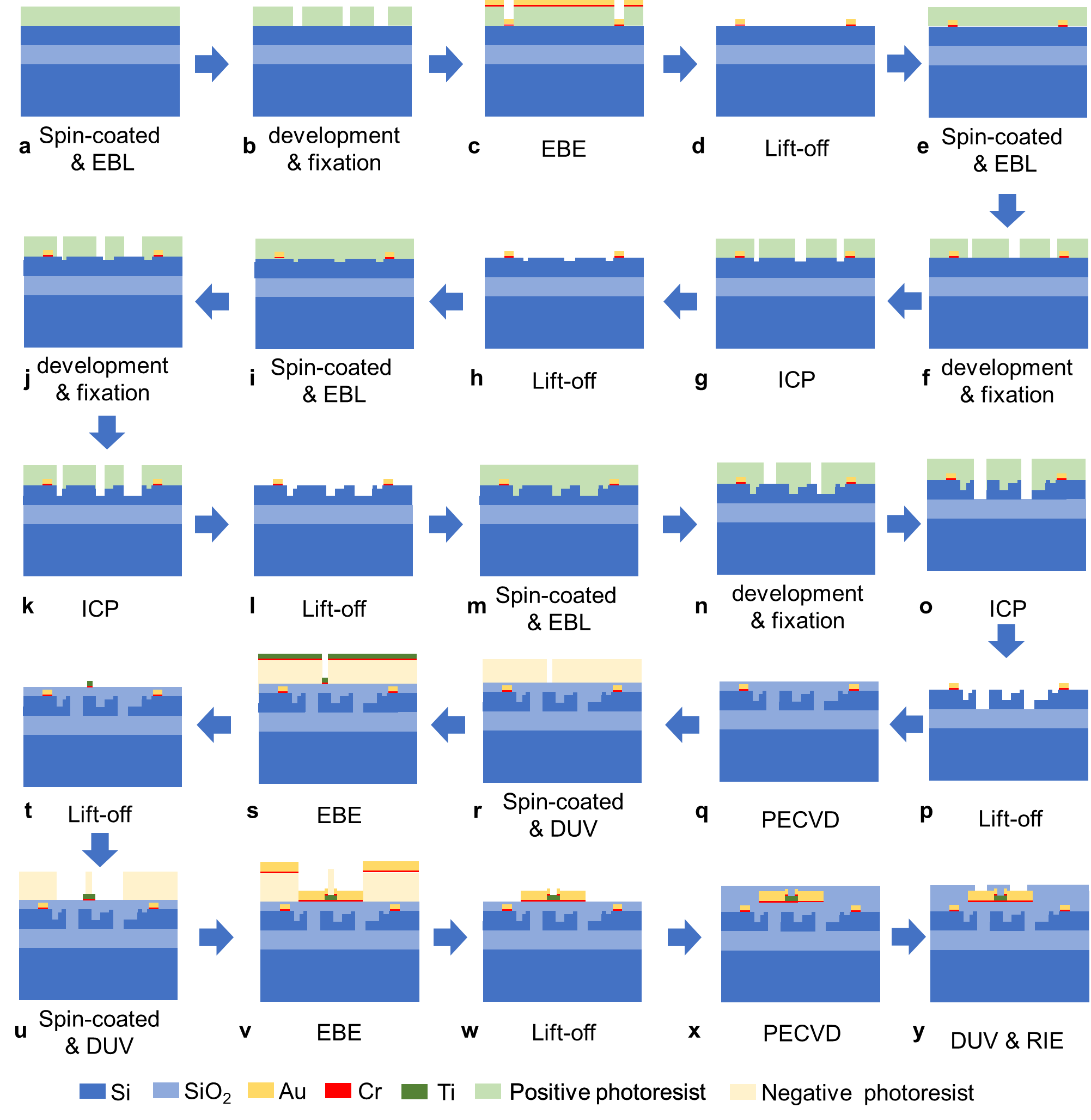


**Fig. S1.** Fabrication process of the silicon photonics chip.

# S3: Femtosecond laser direct writing technique

Fig. S2 shows the schematic of 3D femtosecond laser fabrication technology with a high repetition rate Ti:sapphire oscillator (1030 nm wavelength, 200 kHz repetition rate, 234 fs pulse duration) as the laser source. The generated femtosecond laser beam is reflected by a dichroic mirror and propagates through a linear slit and an objective. A 300 nm linear slit is used to modify the laser beam profile with a 1.5-mm diameter. Then, the linearly polarized femtosecond laser is vertically focused ~50 μm below the top surface of a silica photonics chip through a 50 × 0.42 objective (M Plan Apo NIR, Mitutoyo). The silica chip is placed on a high-precision XY air-bearing stage, providing 2D (x direction and y direction) motion. The z-direction motion is achieved by adjusting the objective. In the 3D fabrication, the scan speed of the femtosecond laser is set to a constant value of 0.2 mm/s, and the pulse energy before the objective is 355 nJ. In addition, the waveguides fabricated with two inscribed traces are used to improve the refractive index and ensure the smoothness of the waveguides. The refractive index contrast of fabricated waveguides is approximately 0.3%. The diameters of single-mode and multi-mode waveguides are chosen as 9 μm and 14 μm, respectively, which can support the low-loss transmission of different modes.


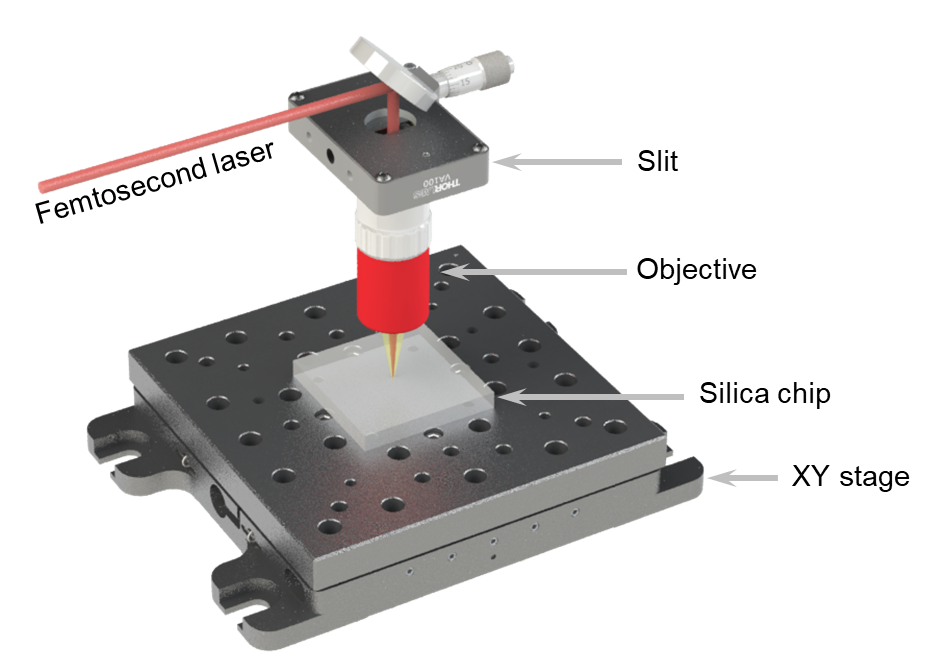


**Fig. S2.** 3D femtosecond laser fabrication technology of the silica photonics chip.

# S4: 3D silica device

1. ***Design***

Fig. S3**a** illustrates the schematic of a photonic lantern device employing a non-uniform waveguide structure, which primarily consists of two parts: Part #1 and Part #2. Part #2 transforms three linearly arranged waveguides into a triangular configuration with a side length of 40 μm. In this process, the central waveguide gradually widens from 9 μm to 11 μm. Part #1 brings the three triangularly arranged waveguides closer together while exciting distinct optical modes. Here, the central 11 μm waveguide tapers back down to 9 μm. Due to the structural asymmetry, the output beams from different waveguides exhibit distinct mode evolution patterns: the central waveguide transitions into a fundamental mode, while the two side waveguides evolve into LP modes that are mirror-symmetric. This asymmetric uniform waveguide path device thus achieves selective excitation and multiplexing of three LP modes. Notably, the device utilizes customized 3D trajectories. Optical waveguides suffer bending loss, an energy attenuation effect caused by curvature variations, similar in principle to fiber bending loss. Transition loss, on the other hand, arises from discontinuities in curvature. By ensuring the continuity of the curve function, near-zero transition loss can be achieved. The curve is defined as follows (see Figure S3**b**):

$$y= \frac{6h}{L^{5}}z^{5}-\frac{15h}{L^{4}}z^{4}+\frac{10h}{L^{3}}z^{3}$$

where *L* and *h* represent the propagation distance and lateral offset, respectively. The 3D trajectory is mapped such that both the XY and YZ planes follow this curve, as depicted in Fig. S3**c**. Fig. S3**d** presents a mode-evolution-based optical transmission simulation of Part #1 of the photonic lantern. The simulation results reveal that the input modes from the two side waveguides evolve in a mirror-symmetric fashion. As a result, one exhibits a +45° orientation while the other shows a -45° distribution, creating the appearance of a 90° mode rotation between the two LP modes.


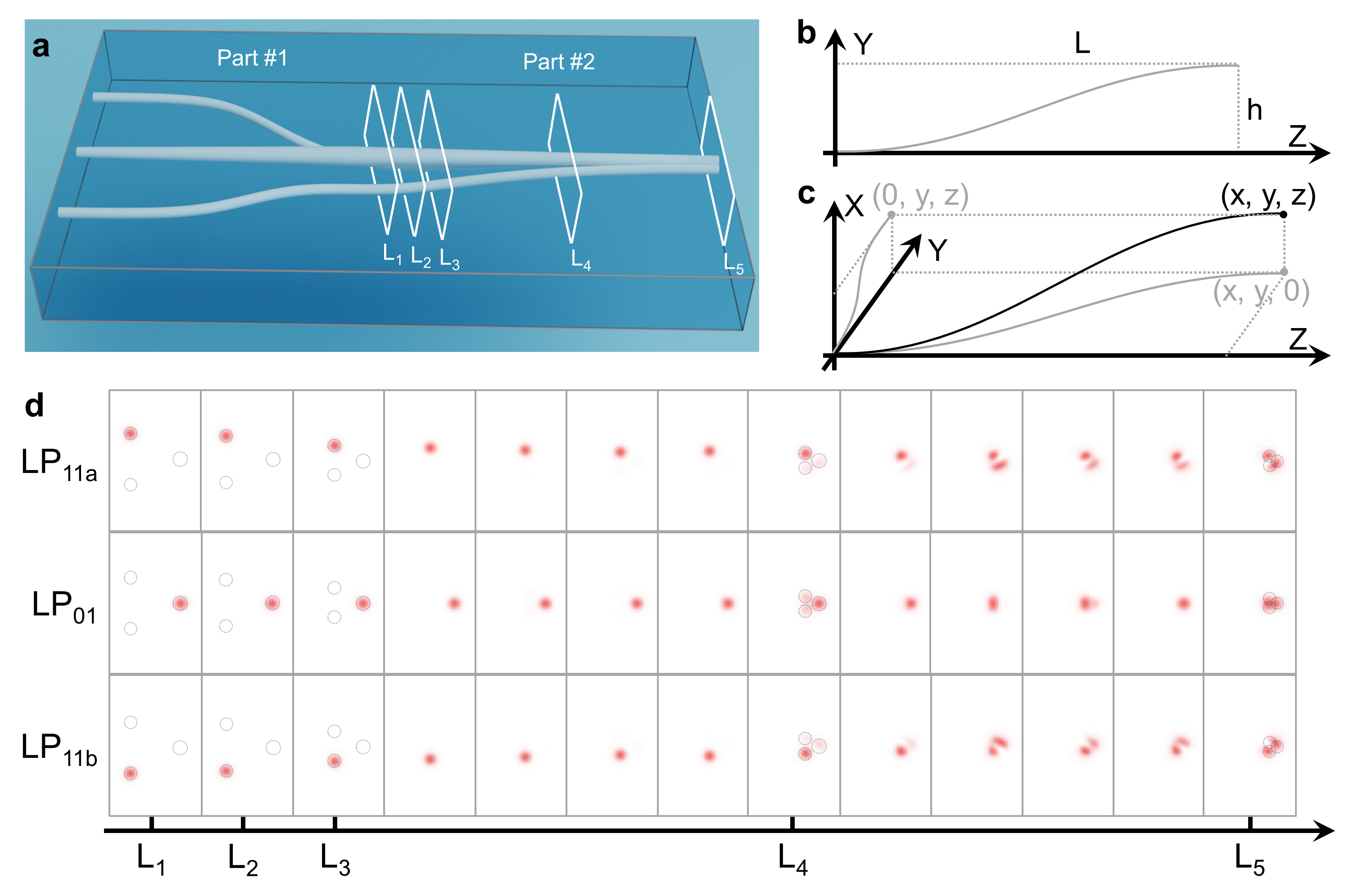


**Fig. S3.** 3D photonic lantern. **a** Schematic structure; Optimized **b** 2D and **c** 3D bending curves; **d** Mode field evolution.

1. ***Repeatability and tolerance***

We fabricated three identical photonic lantern devices in the same batch under identical conditions. The crosstalk matrices (Figs. S4**a**-**c**) indicate consistent performance, with crosstalk levels below -16.5 dB. The total insertion loss for two cascaded devices connected by fibers remains below 3 dB, implying that each device has an insertion loss below 1.5 dB. Therefore, by rearranging the configuration of these three devices, a three-core few-mode fiber can be realized, demonstrating the feasibility of a compact fan-in/fan-out design for multi-core few-mode fibers with more cores. Furthermore, our experiments reveal a linear relationship between femtosecond laser power and waveguide dimensions, as shown in Fig. S5. A 10 mW increase in laser power results in a 1-μm expansion of the waveguide cross-section. Based on this result, we systematically varied the fabrication laser power by ±10 mW to produce devices with controlled dimensional changes. Remarkably, even with these intentional fabrication variations, all devices maintained excellent performance with insertion loss below 2 dB and crosstalk suppression better than -15.5 dB (Figs. S4**d**-**f**). Additionally, we fabricated two devices with different single-mode array port spacings in Part #2: 63.5 μm and 40 μm. Figs. S4**g**-**h** present the crosstalk matrices for both devices, demonstrating insertion loss below 1.5 dB and crosstalk levels under -16.5 dB. These results indicate that the single-mode port spacing does not compromise device performance as long as the spacing remains sufficiently large to prevent inter-port coupling.


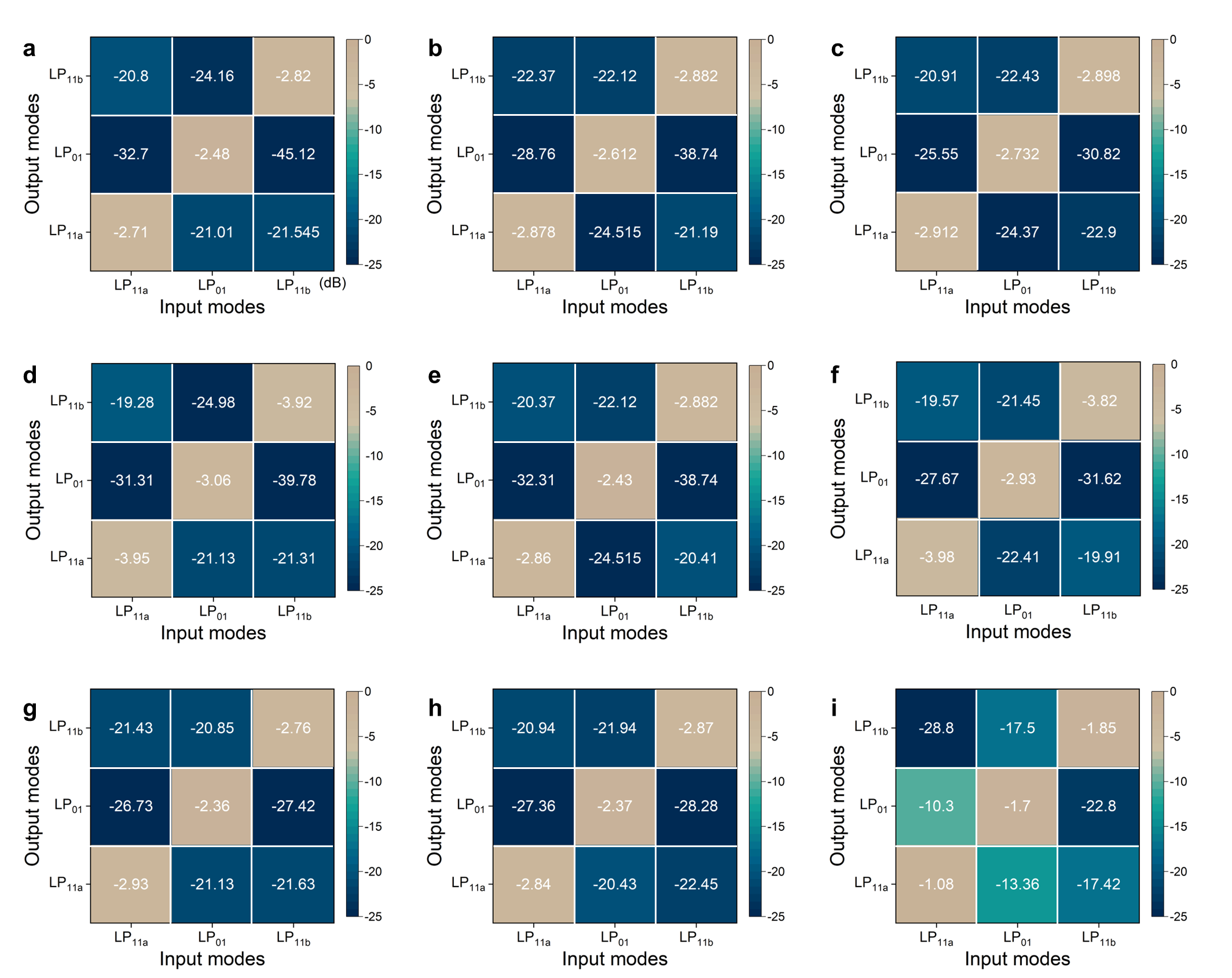


**Fig. S4.** Transmission matrices of 3D photonic lantern chips. **a**-**c** Three devices fabricated in the same batch. **d**-**f** Photonic lantern devices fabricated using femtosecond laser writing with **d** 10 mW lower power, **e** standard power, and **f** 10 mW higher power. **g** Device with 63.5 μm single-mode output port spacing. **h** Device with 40 μm single-mode output port spacing. **i** Commercial photonic lantern device.

1. ***Compare***

Fig. S4**i** displays the crosstalk matrix of the photonic lantern device commercially available from Yangtze Optical Fibre and Cable (YOFC). The device exhibits insertion loss below 1.85 dB for all three modes, with crosstalk levels below -9.22 dB. In comparison, our fabricated device demonstrates superior performance, achieving insertion loss below 1.5 dB and crosstalk better than -16.5 dB for all three modes, significantly outperforming the commercial counterpart.


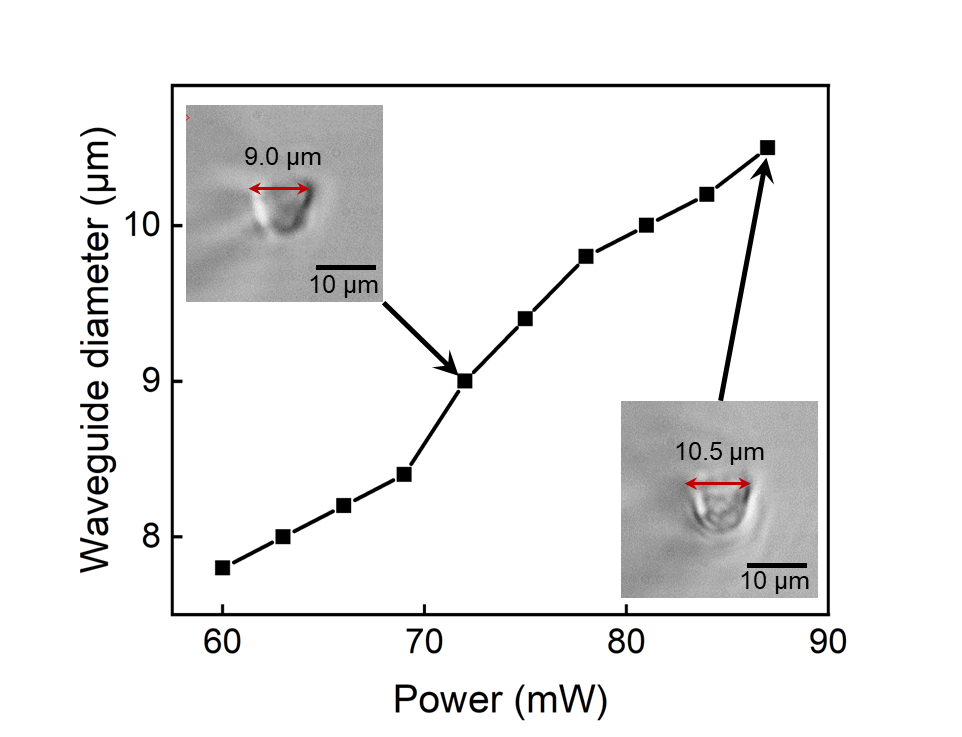


**Fig. S5.** The variation of 3D waveguide size with different femtosecond laser powers.

# S5: Single-mode and multi-mode coupling of the presented hybrid coupler

As shown in the inset of Fig. S6**a**, the single-mode coupling between the silicon waveguide and silica chip employs a silicon adiabatic taper with a length L_tap_ of 160 μm and a tip width W_tip_ of 160 nm for a trade-off between the fabrication process and the coupling efficiency. The single-mode waveguide width is 450 nm. Fig. S6**a** shows the simulated and measured coupling efficiencies. The simulated and measured coupling losses of TE_0_ (TM_0_) mode are less than 0.9 dB (1.5 dB) and 1.6 dB (2 dB), respectively. The simulated and measured results of two polarizations show a discrepancy that may be induced by fabrication errors, fabrication-induced roughness, and test errors.


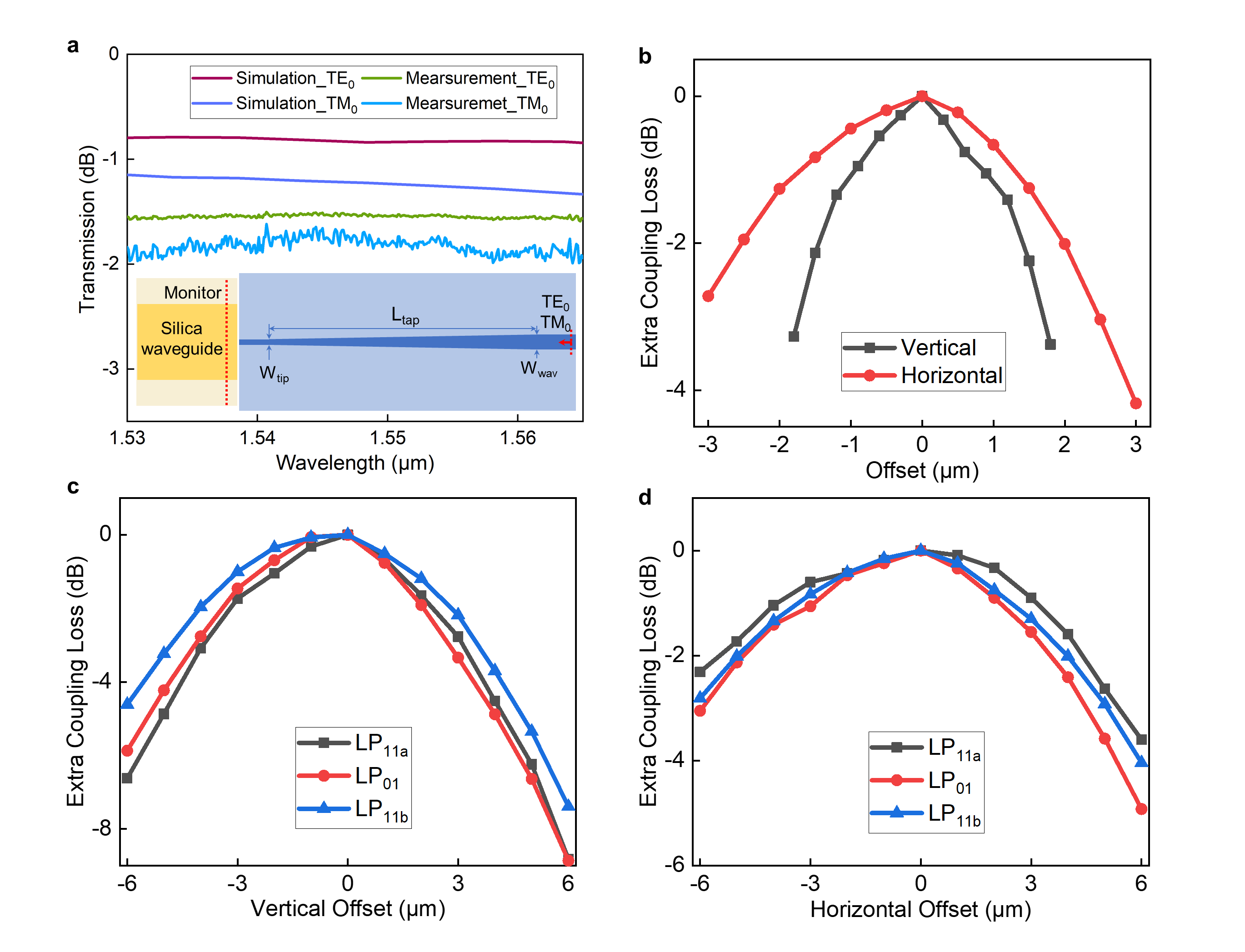


**Fig. S6.** Edge coupling. **a** Coupling efficiency in simulation and measurement for single-mode coupler between silicon chip and silica chip. The inset is the structure diagram of coupler. **b** Extra coupling loss of single-mode waveguide taper for different horizontal offsets and vertical offsets. **c**,**d** Extra coupling loss of few-mode port for different **c** vertical offsets and **d** horizontal offsets.

Fig. S6**b**-**d** show the additional coupling loss as a function of horizontal and vertical offsets. For single-mode coupling using a waveguide taper, the 3-dB alignment tolerance ranges from -3 µm to 2.2 µm (-1.5 µm to 1.4 µm) in the horizontal (vertical) direction. For multi-mode coupling at the interface between the FMF and silica chip, the 3-dB alignment tolerance ranges from -6 µm to 4.5 µm (-3 µm to 3 µm) in the horizontal (vertical) direction. The results indicate that the 3-dB alignment tolerance for multi-mode coupling is greater than that for single-mode coupling, due to the larger mode field area of the multi-mode waveguide. Additionally, for both types of coupling, the 3-dB alignment tolerance in the horizontal direction is larger than that in the vertical direction.

# S6: Silicon polarization handing devices

Polarization management is one of the most important multiplexing technologies. Due to the strong waveguide birefringence, silicon photonic waveguide has a significant refractive index difference, which enables high-performance polarization management devices, including a polarizer, a polarization-beam splitter (PBS), a polarization-splitter-rotator (PSR), and a polarization rotator (PR).

1. ***Polarization beam splitter (PBS)***

PBS can separate/combine two polarization states and preserve the original polarization direction. The PBS employs three cascaded bent directional couplers (DCs #1, #2, and #3). As shown in Fig. S7**a**, DC #2 and DC #3 are connected to two decoupling ports of DC #1. DC #1 and DC #2 have the same design parameters to achieve the cross-coupling of TM_0_ mode and the through-transmission of TE_0_ mode according to the phase matching of TM_0_ mode and the phase mismatching of TE_0_ mode ^1^. Fig. S7**c** displays the simulated light propagation in the silicon PBS for the TE_0_ mode and TM_0_ mode. TE_0_ is routed to the through port, while TM_0_ is routed to the cross port. DC# 2 and DC #3 suppress the TE_0_ mode in the cross port and TM_0_ mode in the through port, respectively, which can reduce the crosstalk of PBS. A microscope photograph of the silicon PBS is shown in Fig. S7**b**. Fig. S7**d** and S7**e** are the measured transmission spectra at the two ports of the decoupling region, which clearly show the insertion loss of < 0.4 dB and < -30 dB crosstalk across the entire C band. The transmittances for TE_0_ and TM_0_ modes exceed 100% in some wavelengths. This may be due to fabrication imperfections leading to unequal coupling losses in devices with and without the PBS.

1. ***Polarization splitter rotator (PSR)***

PSR not only achieves polarization separation but also polarization rotation, making it an attractive device for polarization-related optical systems. As shown in Fig. S8**a**, the PSR consists of a wide ridge tapered waveguide (part #1), a shallow-etch ADC-based mode-selective coupler (part #2), and two narrow ridge tapered waveguides (part #3). Based on mode hybridization and mode coupling, part #1 performs polarization rotation, while part #2 performs polarization separation. Fig. S8**b** and Fig. S8**c** show the simulated light propagation in part #1 (the launched TE_0_ and TM_0_ mode) and part #2 (the launched TE_0_ and TE_1_ mode), respectively. Part #1 can convert the TM_0_ mode to the TE_1_ mode. Then, the TE_1_ mode from the wide tapers can be coupled to the TE_0_ mode of the narrow tapers by part #2. The TE_0_ mode, however, passes through both parts without mode conversion or coupling and is output from the through port. Part #3 connects the ridge waveguide and strip waveguide with negligible loss. Fig. S8**d** and Fig. S8**e** show the transmission spectra of part #1 (the launched TE_0_ mode and TM_0_ mode) and part #2 (the launched TE_0_ mode and TE_1_ mode), respectively. Fig. S9**a** shows the microscope photograph of the silicon PSR. It can be seen that part #1 (part #2) has a low insertion loss of < 0.03 dB (0.07 dB) and a low crosstalk of < -25 dB (-24 dB). Fig. S9**b** and Fig. S9**c** depict the transmission spectra of the fabricated PSR when the TE_0_ mode and TM_0_ mode are launched, showing an insertion loss below 1 dB and crosstalk below -20 dB.

1. ***Polarization rotator (PR)***

PR converts one polarization to the orthogonal one (i.e., TE_0_ → TM_0_ or TM_0_ → TE_0_). In this work, the PR is implemented using the input and cross ports of the PSR above for simplicity and convenience (see Fig. S10).


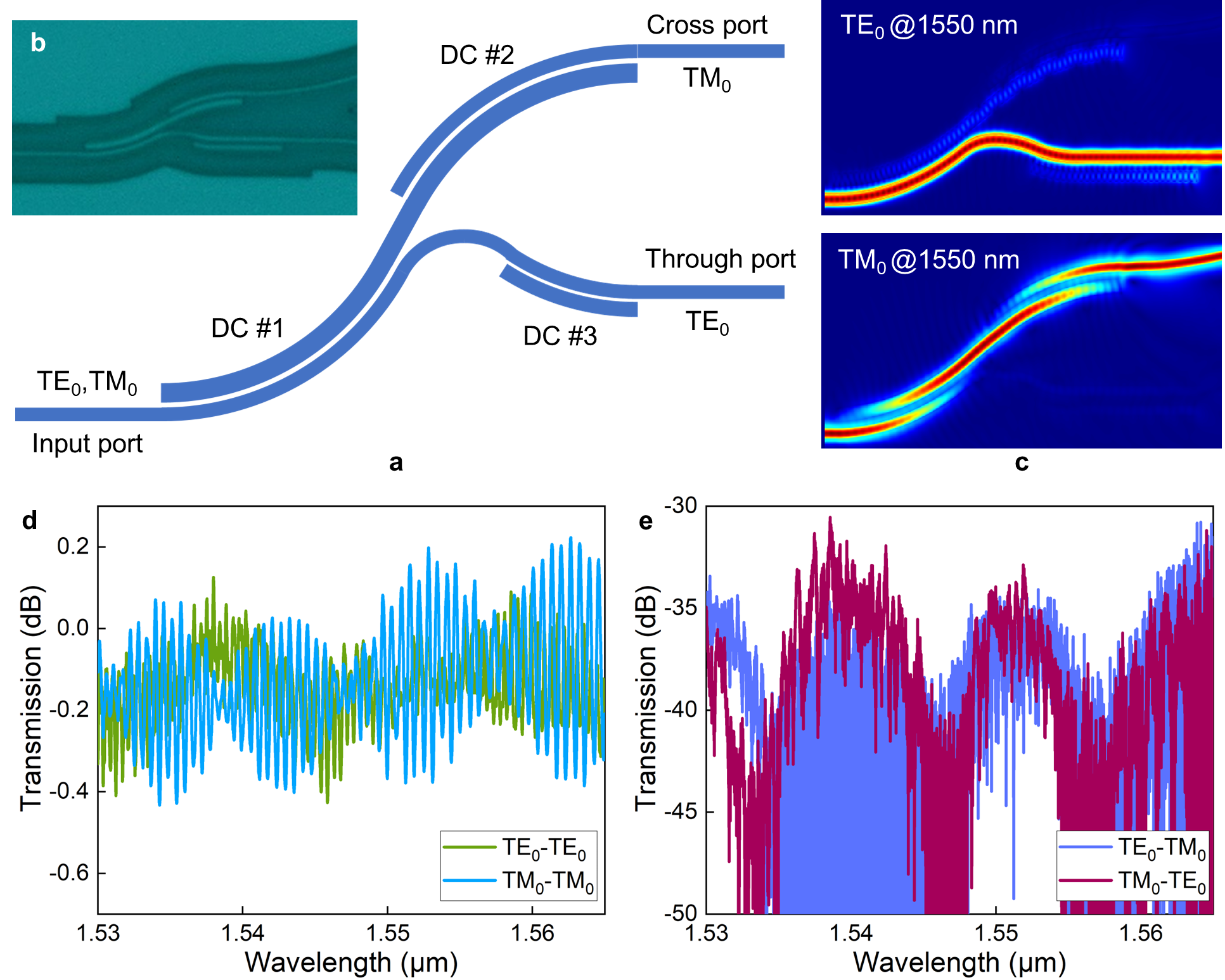


**Fig. S7.** Simulation, fabrication, and measurement of silicon PBS. **a** Schematic diagrams of the silicon PBS. **b** Microscopy image of the fabricated silicon PBS. **c** Simulated field distribution of the designed PBS at 1550 nm wavelength when the TE_0_ and TM_0_ modes are launched. The measured **d** insertion loss and **e** crosstalk of the silicon PBS.


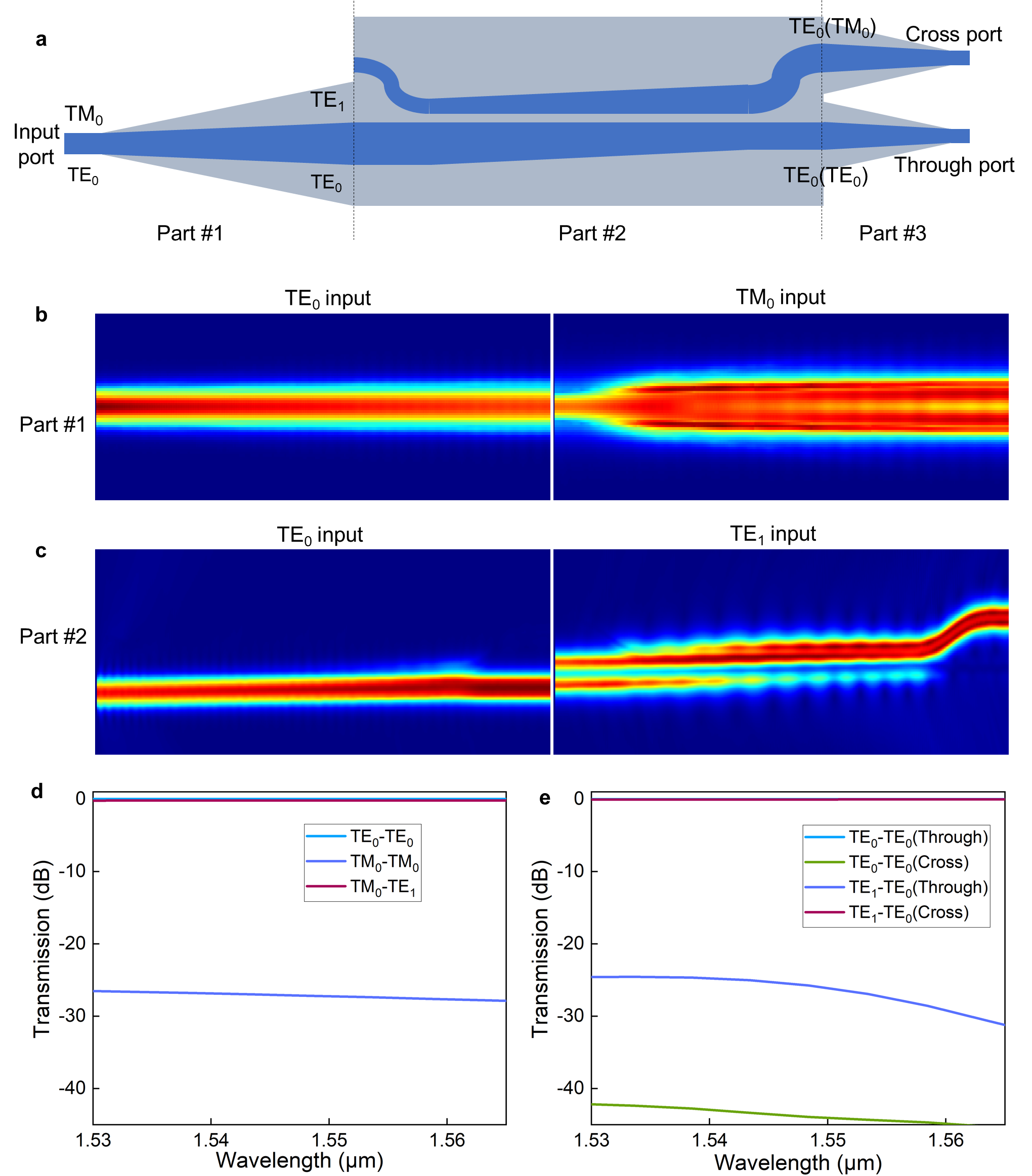


**Fig. S8.** Design and simulation of silicon PSR. **a** Schematic diagrams of the silicon PSR consisting of part #1 and part #2. **b** Simulated field distribution of the designed PSR of the part #1 at 1550 nm wavelength when the TE_0_ and TM_0_ modes are launched. **c** Simulated field distribution of the designed PSR of part #2 at 1550 nm wavelength when the TE_0_ and TE_1_ modes are launched. **d** Simulated transmission spectra of the launched the TE_0_ and TM_0_ modes in part #1. **e** Simulated transmission spectra of the launched the TE_0_ and TE_1_ modes in part #2.


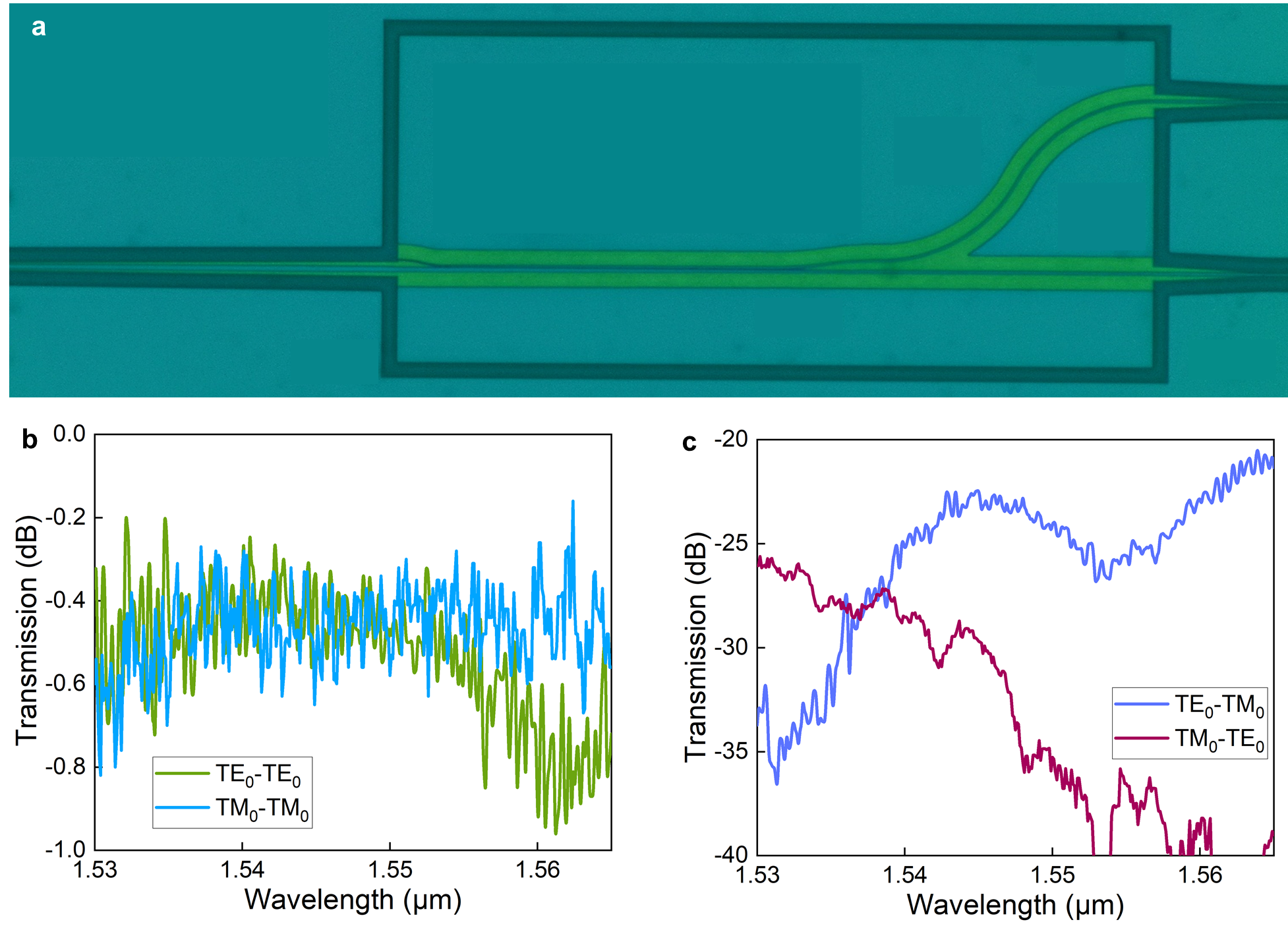


**Fig. S9.** Fabrication and measurement of silicon PSR. **a** Microscopy image of the fabricated silicon PSR. The measured **b** insertion loss and **d** crosstalk of the silicon PSR.


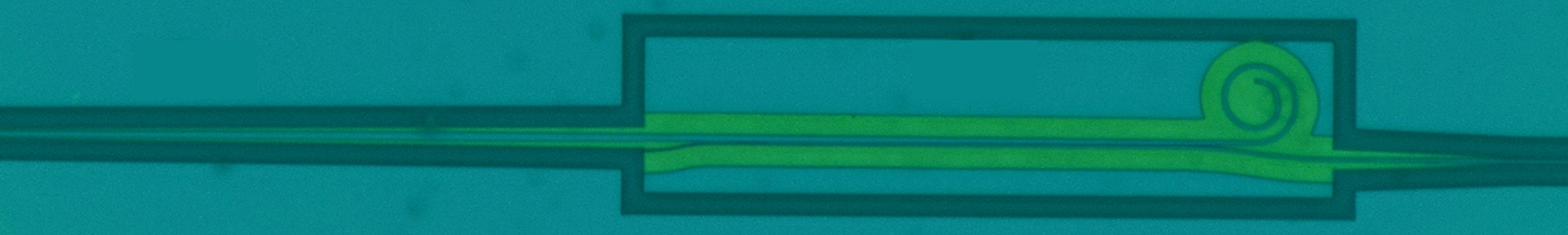


**Fig. S10.** Microscopy image of the fabricated silicon PR.

# S7: Silicon mode multiplexers

The mode demultiplexer uses cascaded silicon asymmetric directional couplers (ADCs) consisting of a narrow waveguide and a wide waveguide, as shown in Fig. S11**a**. The principle is based on mode coupling theory. The high-order mode in the wide waveguide can be excited from or demultiplexed into the fundamental mode in the narrow waveguide when the effective refractive index of the fundamental mode equals that of the high-order mode. Fig. S11**b** and S11**c** show the effective refractive indexes (n_eff_) of 220-nm-thick silicon waveguides with the sidewall angles of 90^◦^ and 85^◦^, respectively. Both show that n_eff_ of TE modes is more sensitive to the waveguide width than that of TM modes. Therefore, the mode/polarization (de)multiplexers use the ADC structures for TM modes and tapered adiabatic ADC structures for TE modes. In addition, the imperfect etching process usually results in unexpected sidewall errors in the fabricated silicon waveguide. In our previous analyses^2,3^, the sidewall error causes the mode hybridization and mode conversion when the width range of the tapered adiabatic waveguide falls within the hybridization region (see the red dashed box in Fig. S11**c**) and the introduced polarization crosstalk in tapered adiabatic ADC structure. To solve the problem, the ADC structures with a short coupling length or a width variation range avoiding the mode hybridization region are considered in the design of mode/polarization (de)multiplexers. The simulated field distributions of the designed couplers for the TM_2_, TM_1_, TE_2_, and TE_1_ modes are shown in Fig. S11**d-**Fig.S11**g**, respectively, when the operating wavelength is 1550 nm. The six modes in the silicon multi-mode waveguide can all be coupled to the TE_0_ or TM_0_ mode in different waveguides. Fig. S12 reports the transmission spectra of these ADC-based mode demultiplexers, which show that the insertion losses for TM_2_, TM_1_, TE_2_, and TE_1_ modes are less than 0.35 dB, 0.2 dB, 0.04 dB, and 0.04 dB and that the crosstalk is less than −25 dB in the wavelength range of 1530-1565 nm. The microscopy image of the fabricated six-mode (de)multiplexer includes four ADC structures and a PBS, as shown in Fig. S13**a**. The measurement transmission results are shown in Fig. S13**b**. It shows low excess loss (< 1 dB) and low crosstalk (-16 dB) over the C-band (1530-1565 nm).


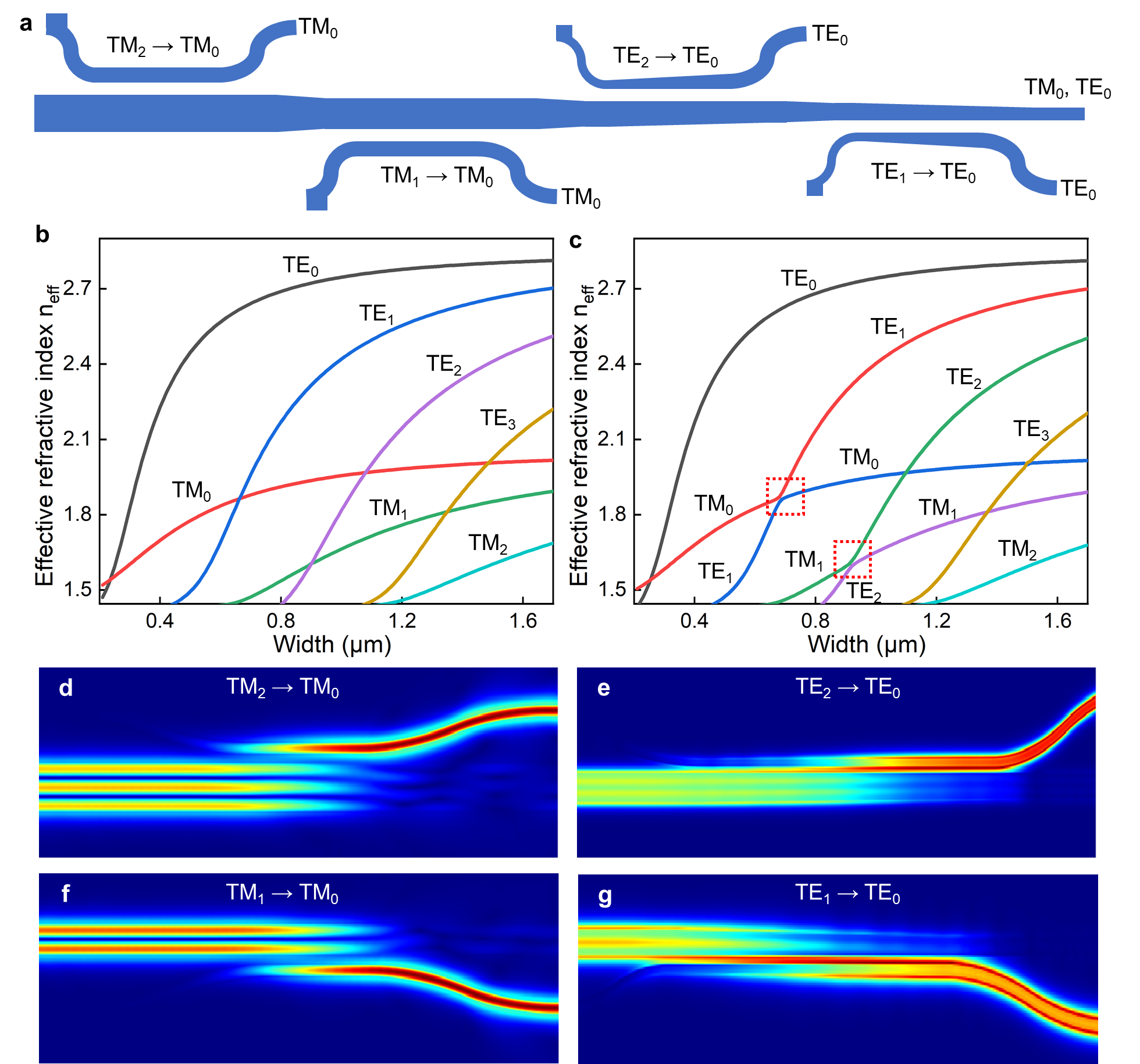


**Fig. S11.** Design of cascaded ADC-based mode multiplexer. **a** Schematic diagrams of the cascaded ADC-based structures. Calculated mode effective index (n_eff_) for the silicon photonic waveguides with **b** 90◦ sidewalls and **c** 86◦ sidewalls. The simulated field distribution of the designed couplers for the **d** TM_2_, **e** TE_2_, **f** TM_1_, and **g** TE_1_ modes at 1550 nm wavelength.


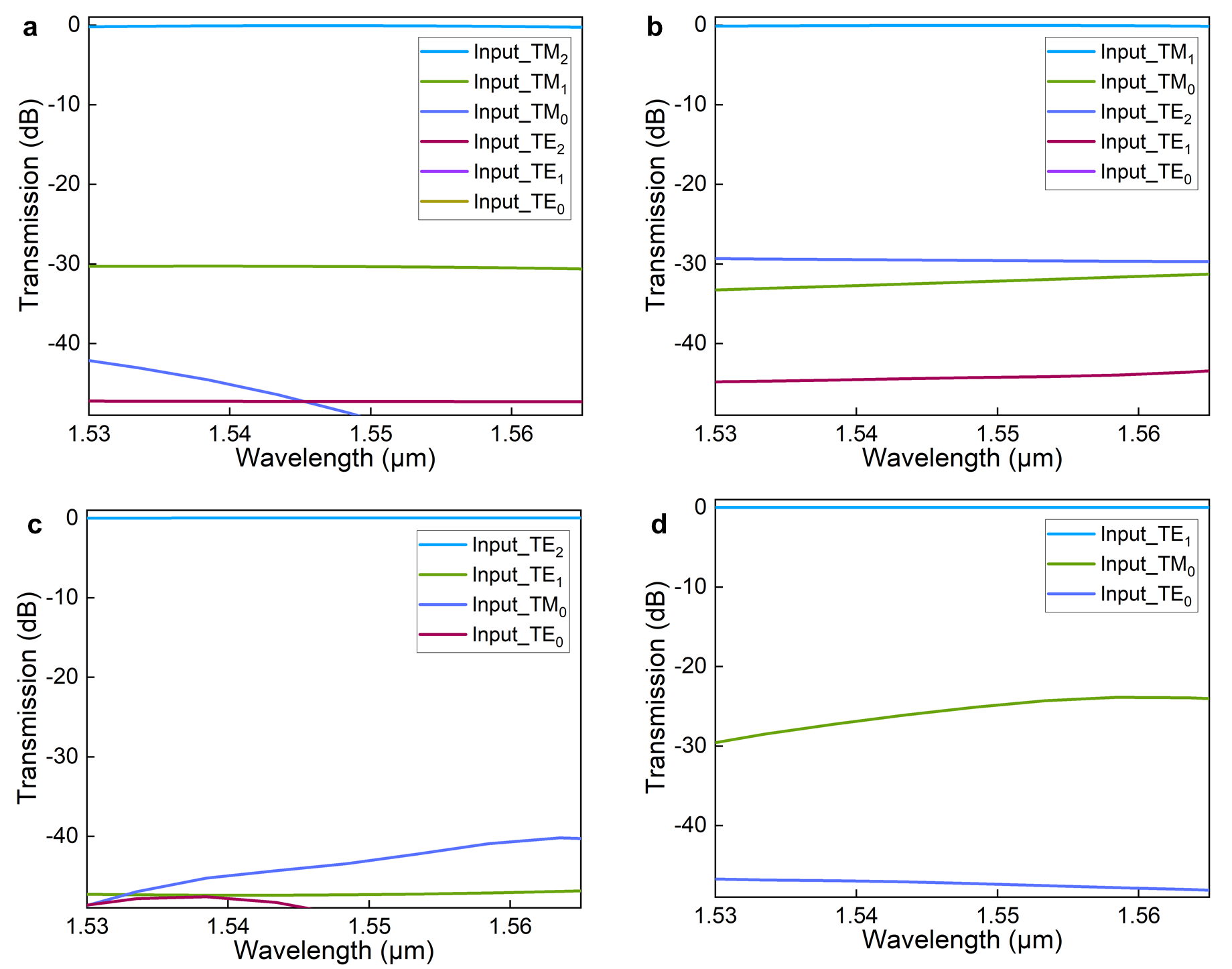


**Fig. S12.** Simulated transmission spectra of the launched different modes in the corresponding **a** TM_2_, **b** TM_1_, **c** TE_2_, and **d** TE_1_ coupler in the wavelength range of 1530-1565 nm.


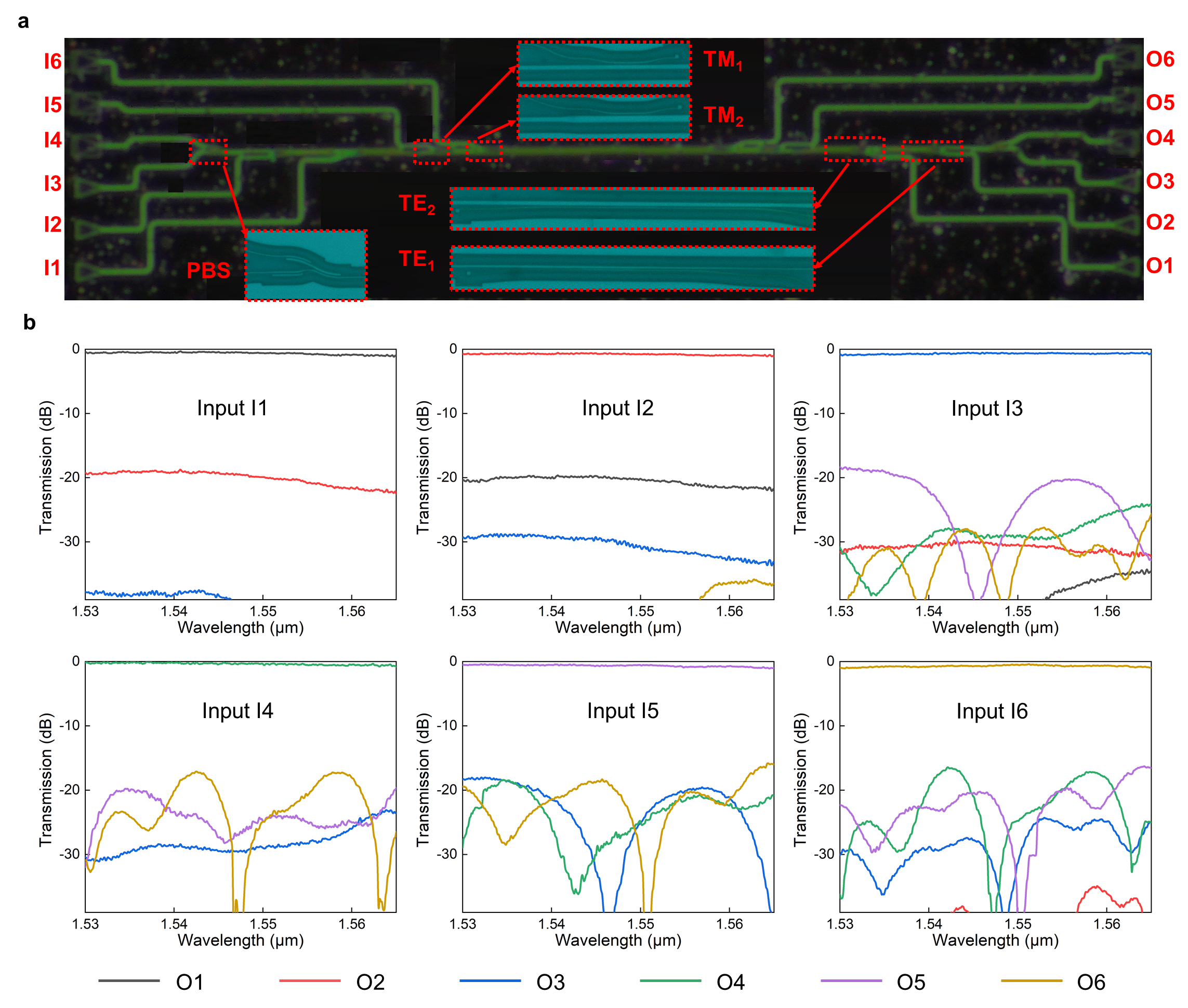


**Fig. S13.** **a** Microscopy image and enlarged image of the fabricated silicon mode (de)multiplexer. **b** the measured transmission spectra of 6 out ports of the silicon mode (de)multiplexer in the wavelength range of 1530-1565 nm, when the light is injected to the six input ports one by one.

# S8: Measurement of the inter-modal crosstalk for the multi-mode coupler

Fig. S14 shows the experimental configuration for measuring the inter-modal crosstalk of the hybrid 2D/3D integrated coupler, which includes a wavelength-tunable laser, polarization controller (PC), the LP mode (de)multiplexers, an optical switch, the multi-mode coupler, and multiple power meters. The LP mode (de)multiplexers, consisting of multiple fiber-based PBSs and a silica mode-selective coupler, are used to excite different LP modes in FMF. The modes in the multi-mode waveguide (MMW) are monitored by the silicon mode multiplexer. In the hybrid integration method, the silica chip and the few-mode fiber (FMF) are permanently secured using encapsulation technology. For coupling between the silica and silicon chips, the silicon chip is fixed on a chip mounting stage. Precise alignment of the silica chip is achieved through a six-axis adjustment platform. To facilitate fiber handling by the six-axis platform, a custom-made FMF fiber array is employed to enable coupling through the encapsulation and curing process. The wavelength-tunable laser can generate light with different wavelengths, while multiple power meters can simultaneously monitor optical power. During measurement, six output ports are recorded simultaneously by six power meters, as the light is injected into the different input ports of the LP mode multiplexer one by one. As illustrated in Fig. S15, the measured inter-modal crosstalk of the hybrid 2D/3D integrated coupler at different wavelengths can be obtained by tuning the wavelength of the laser. We selected eight wavelengths with 5-nm spacing, covering the whole C band. The measured results indicate that all inter-modal crosstalk levels are below -15 dB.


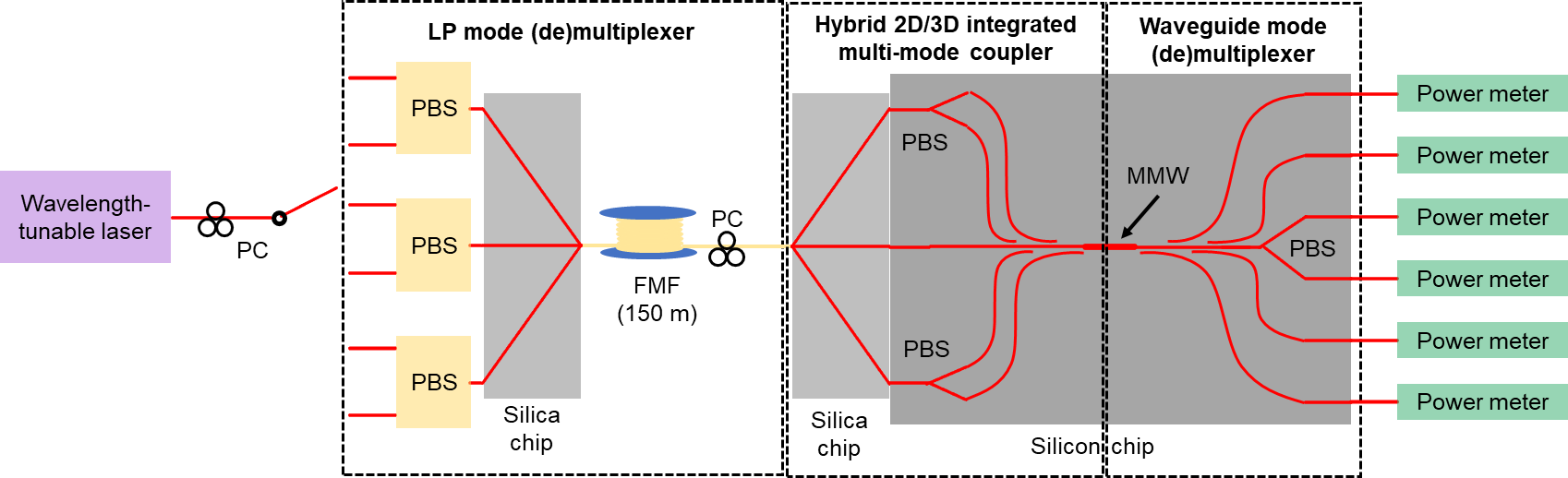


**Fig. S14.** Experiment setup for measuring the inter-modal crosstalk for the hybrid 2D/3D integrated coupler.


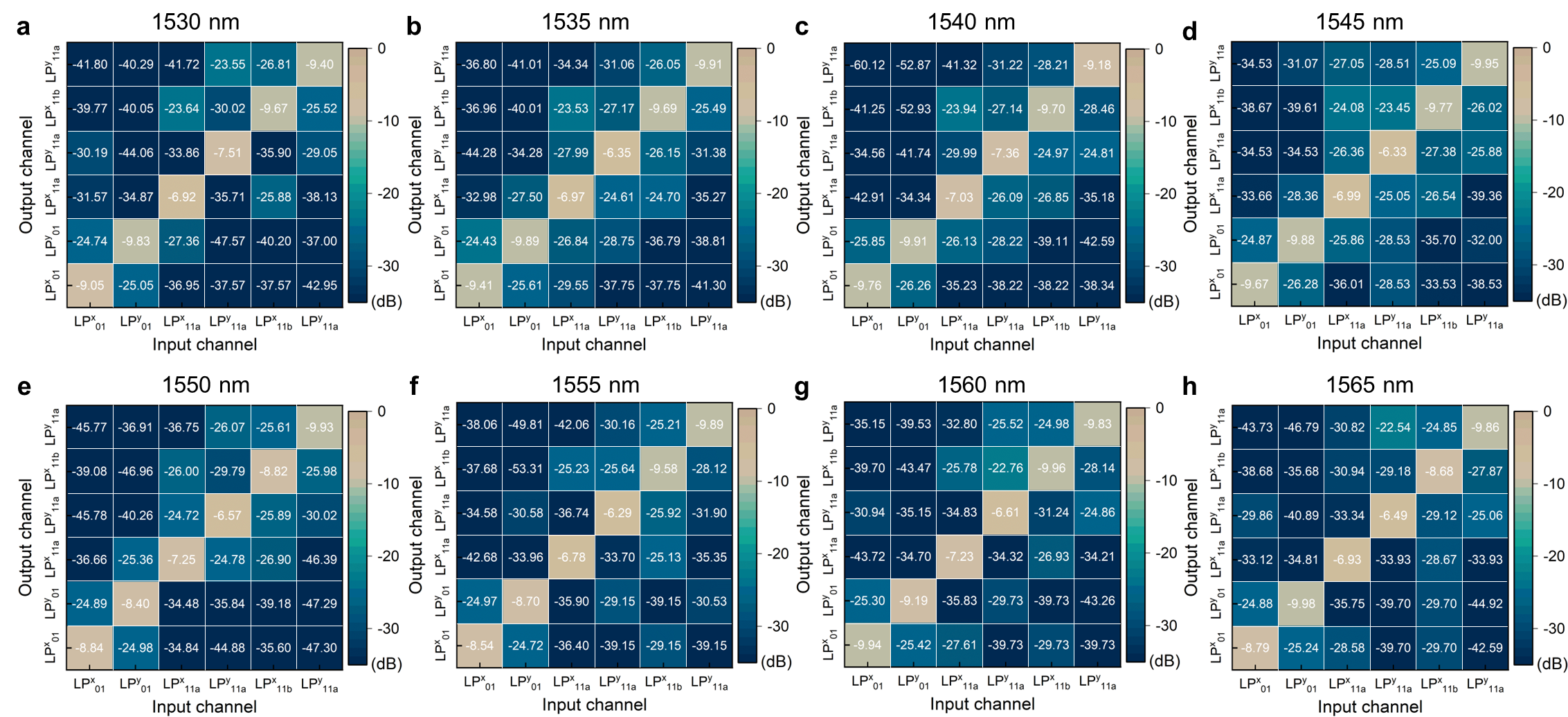


**Fig. S15**. Inter-modal crosstalk for the hybrid 2D/3D integrated coupler at the wavelengths of **a** 1530 nm, **b** 1535 nm, **c** 1540 nm, **d** 1545 nm, **e** 1550 nm, **f** 1555 nm, **g** 1560 nm, and **h** 1565 nm.

# S9: MRR thermal crosstalk

Fig. S16 shows the wavelength shifts of the other 31 tuned center wavelengths when the voltage applied to the MRR for the first wavelength is varied from 0 V to 4.5 V. The observed wavelength deviations range from -0.07 nm to +0.08 nm, with positive (red shift) and negative (blue shift) values indicating shifts in the center wavelength. From the spectral profiles at the drop port, a maximum wavelength shift of 0.08 nm introduces approximately 2 dB of additional crosstalk. Moreover, to mitigate wavelength drift, a feedback control loop is implemented to stabilize the center wavelengths. Therefore, the thermal crosstalk caused by different voltages has a minimal impact on our experimental performance.


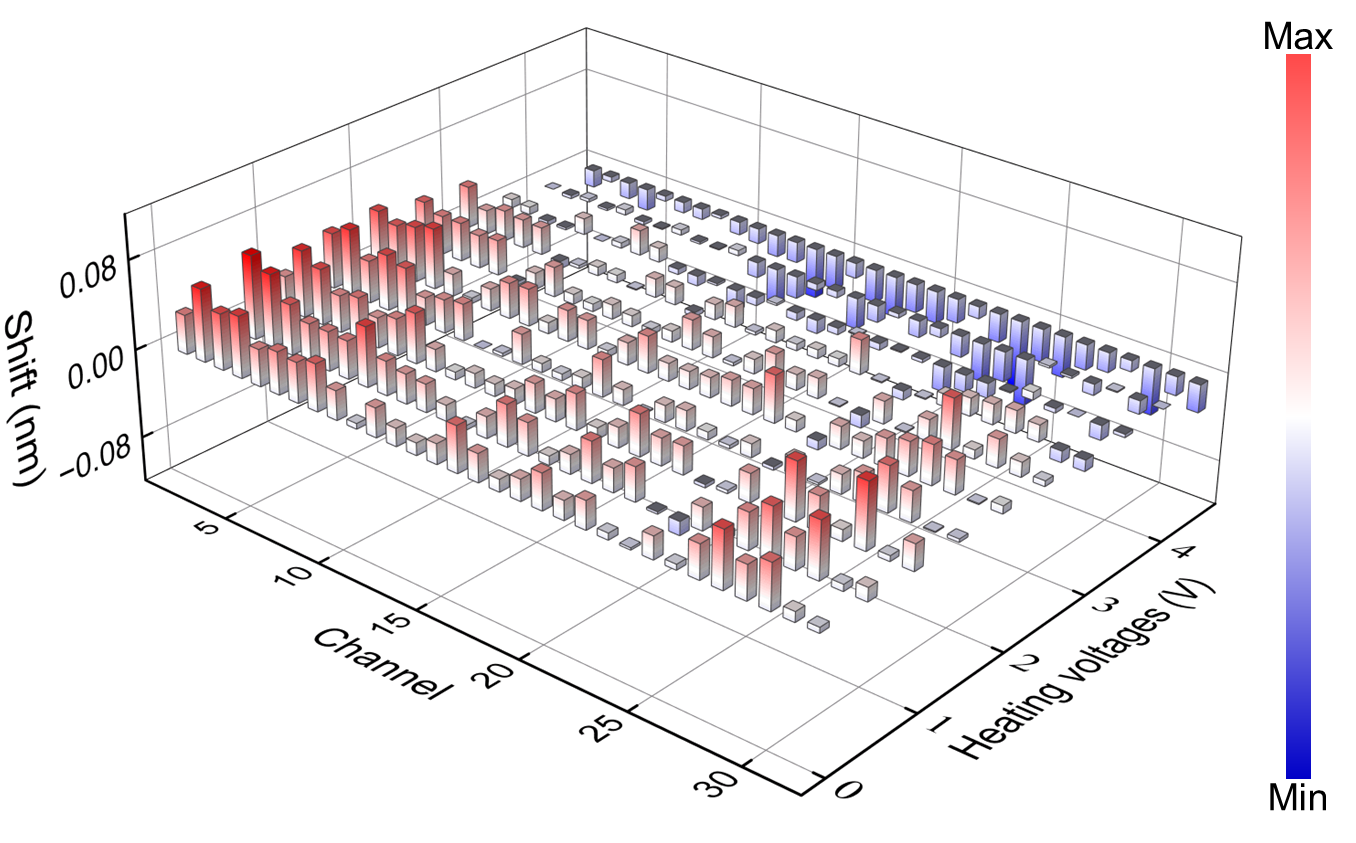


**Fig. S16.** As the voltage of the MRR corresponding to the first wavelength is adjusted from 0 V to 4.5 V, the remaining 31 tuned center wavelengths exhibit corresponding shifts.

# S10: Comparisons of different kinds of integrated chip and fiber-chip systems

In this section, the detailed parameters of the reported high-capacity multi-channel integrated chip based on the multiplexing technology of different dimensions, in recent works are summarized, as shown in Table S2. Furthermore, Table S3 presents a comprehensive comparison of the state-of-the-art fiber-chip systems with few-mode fiber.

**Table S2** Comparison of the high-capacity multi-channel silicon chips.

| Devices | Multi-dimensional multiplexing | Data rate [bit/s] | Number of channels | Functions | Wavelength (nm) | Ref.  (year) |
| --- | --- | --- | --- | --- | --- | --- |
| Microring | WDM, MDM | 60 G | 6 | Data transmission | 1532, 1546, 1563 | [18]  (2014) |
| ADC | MDM, PDM, WDM | 600 G | 20 | Data transmission | 1546.92, 1554.13 | [17]  (2018) |
| ADC | MDM, PDM | 2.6 T | 42 | Data transmission | 1520 - 1560 | [79]  (2018) |
| AWG | WDM | 640 G | 16 | Data transmission  add/drop | 1546.66 - 1558.23 | [41]  (2020) |
| Metamaterial | MDM | 813 G | 8 | Data transmission | ~ 1538.2 | [80]  (2022) |
| ADC, Microring | MDM, PDM, WDM | 960 G | 96 | Data transmission  add/drop | 1560 -1595 | [81]  (2022) |
| ADC with SWG | MDM | 2.162 T | 16 | Data transmission | 1550.12 | [15]  (2023) |
| **ADC, Microring** | **MDM, PDM, WDM** | **20.01 T** | **192** | **Data transmission**  **add/drop** | **1530 - 1555** | **This work** |

AWG: array waveguide grating.

The associated reference labels are from the Ref. [15, 17, 18, 41, 79-81] of the main text.

**Table S3** Comparison of the state-of-the-art fiber-chip systems with few-mode fiber.

| Types | Data rate [bit/s] | Multi-dimensional multiplexing | Number of channels | Multi-mode coupling schemes | Wavelength (nm) | Ref.  (year) |
| --- | --- | --- | --- | --- | --- | --- |
| Chip-fiber-chip | 160 G | MDM | 4 | 2D grating coupler | 1542.1 – 1557.1 | [48]  (2020) |
| Chip-fiber-chip | 400 G | MDM | 2 | 1 × 3 MMI,  triple-tip taper | 1520 - 1610 | [65]  (2020) |
| Fiber-chip | 192 G | MDM | 4 | 2D grating coupler  4 × 4 MMI | 1570 | [55]  (2020) |
| Chip-fiber-chip | 4.36 T | MDM, PDM, WDM | 98 | 2D grating coupler | ~ 1538 - 1565 | [82]  (2022) |
| Chip-fiber-chip | 1.12 T | MDM, WDM | 28 | Inverse-designed coupler | 1540 - 1565 | [49]  (2022) |
| Fiber-chip | 256 G | MDM, PDM, WDM | 16 | 3D polymer taper | 1570 - 1582 | [62]  (2022) |
| Chip-fiber-chip | 580.8 G | MDM | 4 | (Si-SiN) coupler | 1550.12 | [50]  (2023) |
| **Fiber-chip** | **20.01 T** | **MDM, PDM, WDM** | **192** | **Hybrid integrated coupler** | **1530 - 1555** | **This work** |

MMI: multi-mode interferometer.

The associated reference labels are from the Ref. [48-50, 55, 62, 65, 82] of the main text.

# References

1. Wu, H., Tan, Y. & Dai, D. Ultra-broadband high-performance polarizing beam splitter on silicon. *Opt. Express* **25**, 6069 (2017).

2. Li, K., Cao, X., Wan, Y., Wu, G. & Wang, J. Fundamental analyses of fabrication-tolerant high-performance silicon mode (de)multiplexer. *Opt. Express* **30**, 22649 (2022).

3. Dai, D. & Mao, M. Mode converter based on an inverse taper for multimode silicon nanophotonic integrated circuits. *Opt. Express* **23**, 28376 (2015).
